# Slow thermal equilibration in methylammonium lead iodide revealed by transient mid-infrared spectroscopy

**DOI:** 10.1038/s41467-018-05015-9

**Published:** 2018-07-18

**Authors:** Peijun Guo, Jue Gong, Sridhar Sadasivam, Yi Xia, Tze-Bin Song, Benjamin T. Diroll, Constantinos C. Stoumpos, John B. Ketterson, Mercouri G. Kanatzidis, Maria K. Y. Chan, Pierre Darancet, Tao Xu, Richard D. Schaller

**Affiliations:** 1 Center for Nanoscale Materials, Argonne National Laboratory, 9700 South Cass Avenue, Lemont, IL 60439 USA; 2 Department of Chemistry and Biochemistry, Northern Illinois University, 1425W. Lincoln Hwy., DeKalb, IL 60115 USA; 3 Department of Chemistry, Northwestern University, 2145 Sheridan Road, Evanston, IL 60208 USA; 4 Department of Physics and Astronomy, Northwestern University, 2145 Sheridan Road, Evanston, IL 60208 USA

## Abstract

Hybrid organic–inorganic perovskites are emerging semiconductors for cheap and efficient photovoltaics and light-emitting devices. Different from conventional inorganic semiconductors, hybrid perovskites consist of coexisting organic and inorganic sub-lattices, which present disparate atomic masses and bond strengths. The nanoscopic interpenetration of these disparate components, which lack strong electronic and vibrational coupling, presents fundamental challenges to the understanding of charge and heat dissipation. Here we study phonon population and equilibration processes in methylammonium lead iodide (MAPbI_3_) by transiently probing the vibrational modes of the organic sub-lattice following above-bandgap optical excitation. We observe inter-sub-lattice thermal equilibration on timescales ranging from hundreds of picoseconds to a couple of nanoseconds. As supported by a two-temperature model based on first-principles calculations, the slow thermal equilibration is attributable to the sequential phonon populations of the inorganic and organic sub-lattices, respectively. The observed long-lasting thermal non-equilibrium offers insights into thermal transport and heat management of the emergent hybrid material class.

## Abstract (web summary)

Hybrid organic-inorganic perovskites are emerging materials for efficient photovoltaics; however understanding how charge/heat dissipates inside the material remains a challenge. Here, the authors use a spectroscopic approach to observe unusually slow thermal equilibration between the organic and inorganic components.

## Introduction

Solution processable hybrid organic–inorganic perovskites (HOIPs) such as methylammonium lead iodide (MAPbI_3_) represent a research forefront owing to prospects of enhanced performance in solar energy conversion^1^, solid-state lighting^2^, hard radiation detection^3^, and information processing applications^4^. Both HOIPs and their all-inorganic counterparts exhibit superior optoelectronic properties including long carrier diffusion lengths^5^, long carrier lifetimes^6^, and excellent defect tolerance^7^. In comparison to the all-inorganic perovskites which primarily use Cs^+^ as the A-site cation, HOIPs with organic A-site cations have been shown to exhibit enhanced photoluminescence (PL) properties^8^ and protection of hot carriers^9,10^. Incorporation of organic cations also add structural variety, and with it a large design space for optimization of the energy-conversion efficiency and phase stability of perovskite-based optoelectronic devices^11^. The heterogeneity in the atomic masses of the organic and inorganic sub-lattices highlights a unique feature of HOIPs as compared to the all-inorganic counterparts. In particular, the two sub-lattices were thought to be only weakly coupled^12^. Better understanding of the fate of energetic carriers and non-equilibrium phonons in HOIPs is crucial for the further improvement of HOIP-based technologies.

Static and transient experiments based on electronic probing (i.e., around bandgap probing) have been exploited to investigate carrier and structural dynamics of HOIPs^10,13–16^. However, the near-bandgap, transient electronic response, which reveals energy dissipation by hot electrons and holes, does not directly convey information regarding heating of the atoms, or the sub-lattice response to photoexcitation. Direct probing of lattice temperature can provide insights into the mechanisms and timescales of electron–phonon and phonon–phonon interactions.

Herein, we employ visible-pump, mid-infrared-probe transient absorption spectroscopy to investigate lattice heating in MAPbI_3_ and formamidinium lead iodide (FAPbI_3_). The strong temperature sensitivity of the absorbance of the organic sub-lattice vibrational modes permits the probing of lattice thermalization with tens-of-femtosecond time resolution. We demonstrate long thermal equilibration time (hundreds-of-picoseconds to a couple of nanoseconds), that is one to two orders-of-magnitude slower than those observed for inorganic semiconductors. We show that the slow thermal equilibration in HOIPs, which can impact the electronic and heat transport properties of HOIPs, arises from a weakly spectrally overlapping phonon density of states (phDOS). Our study provides insights to manipulate such properties for the broader class of organic–inorganic hybrid materials.

## Results

### Static response of MAPbI_3_ in the infrared

**Fig. 1:**
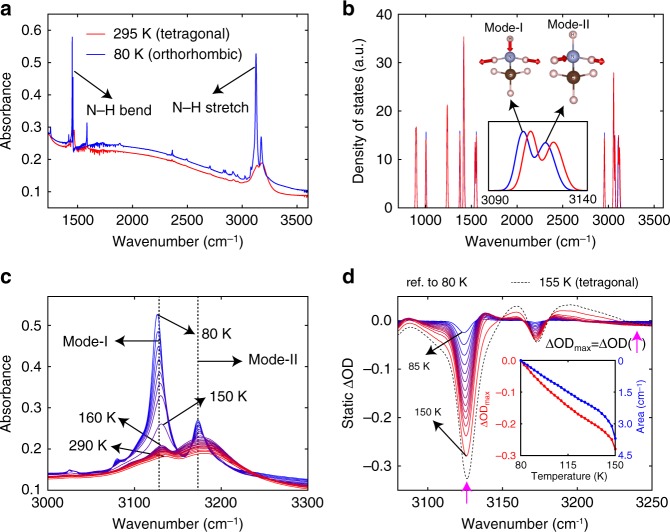
Static infrared absorption spectra of a 680-nm thick MAPbI_3_ film measured by Fourier-transform infrared spectroscopy (FTIR). **a** Experimental infrared absorbance at 295 K (tetragonal phase) and 80 K (orthorhombic phase). **b** First-principles calculated phonon density of states (phDOS) of the orthorhombic phase of MAPbI_3_ (blue: lattice constant at 0 K; red: lattice constant expanded by 0.5%). Inset illustrates the asymmetric N–H stretching modes (mode-I and mode-II). **c** Experimental temperature dependent absorbance of mode-I and mode-II (in increments of 10 K). **d** Differential absorption spectra of mode-I and mode-II from 85 K to 155 K (in increments of 5 K) referenced to 80 K. Inset shows the temperature dependent ΔOD_max_ (defined as the value of ΔOD at the negative peak of mode-I, as indicated by the magenta arrow) and area of ΔOD (defined as the product of ΔOD_max_ and the full-width-half-maximum of the ΔOD dip of mode-I), both referenced to 80 K

Figure 1a shows the infrared absorbance of a 680-nm thick MAPbI_3_ film (see Supplementary Fig. 1 and Supplementary Note 1 for the film thickness determination) in the tetragonal (295 K) and orthorhombic (80 K) phases, which reveal phonon absorption by the MA^+^ cations. The two strong peaks centered between 3100 and 3200 cm^−1^ are assigned to the N–H stretching modes which exhibit strong light-induced changes in dipole moments^17,18^. For the orthorhombic phase of MAPbI_3_ (Fig. 1b and Supplementary Fig. 2), these two strong peaks arise from the asymmetric N–H stretching motions with the lower and higher-frequency modes denoted as mode-I and mode-II, respectively (Fig. 1b inset). Temperature-dependent absorption measurements (Fig. 1c) show that the oscillator strengths for both modes increase at the tetragonal-to-orthorhombic phase transition, where the rotational motions of MA^+^ freeze out^19^. The absorption of mode-I exhibits a stronger temperature dependence in the orthorhombic phase (in comparison to that in the tetragonal phase), and increases nearly linearly with decreasing temperature. These behaviors, analogous to the absorbance of O–H-stretching modes in ice and hydrated salts that increases upon cooling and change discontinuously at a liquid-to-solid phase transition^20,21^, are governed by the strength of hydrogen bonds ^22,23^, here being N–H···I. Specifically, starting at high temperature, no apparent changes in the absorbance of mode-I and mode-II are observed at the cubic-to-tetragonal transition (Supplementary Fig. 3). Their oscillator strengths then grow marginally while they also exhibit small redshifts upon cooling in the tetragonal phase, when the free rotations of MA^+^ hinder the formation of strong hydrogen bonds. In the orthorhombic phase, both modes I and II with significant temperature dependence further exhibit slight redshifts upon cooling, which is a signature of the formation of stronger hydrogen bonds^22^ and is reproduced from 0-K phDOS calculations using slightly different lattice constants that emulate the variation of hydrogen bond strength (Fig. 1b inset).

Earlier first-principles calculations showed that rotations of N–H around the C–N axis in the orthorhombic phase of MAPbI_3_ are energetically unfavorable due to the formation of strong hydrogen bonds^24^. In addition, temperature dependent neutron diffraction experiments performed on the orthorhombic phase of MAPbI_3_ revealed significantly decreased anisotropic displacement parameters of H and moderately decreased anisotropic displacement parameters of I upon cooling^19,25^, and hence a decrease of the time-averaged distance of H···I. Therefore, the increase of hydrogen bond strength, and with it the enhanced absorbance of N–H-stretching-modes is primarily due to the more suppressed thermal motions of H and I, as well as the more restricted tilting of Pb-I octahedra^26^. The static differential absorption spectra referenced to 80 K (Fig. 1d) shows that increasing the temperature results in strong bleaching (i.e., reduction in absorption) of mode-I and weak bleaching of mode-II. Based upon the static temperature dependence, transient absorption (TA) measurements of these two modes should permit the probing of lattice temperature with tens-of-fs time resolution.

### Transient response of MAPbI_3_ in the infrared

**Fig. 2:**
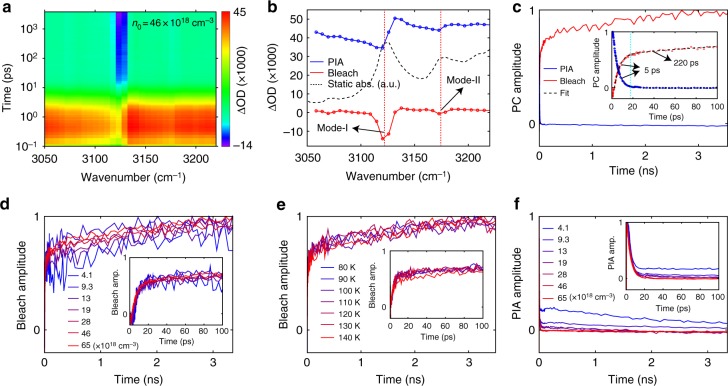
Transient absorption measurements of MAPbI_3_. **a** Transient spectral map of ΔOD measured at 80 K using 500-nm pump excitation with 329 µJ cm^−2^ fluence (corresponding to *n*_0_ of 46 × 10^18^ cm^−3^). The color-coded quantity is ΔOD (×1000). **b** Two principle components (PCs) of the transient response shown in red (bleaching) and blue (photo-induced absorption, or PIA). Circles on the blue and red lines correspond to the pixels of the array detector. The black-dashed line shows the absorbance measured with the array detector without pump excitation. **c** Kinetics of the two PCs up to 3.5 ns. Inset shows the kinetics from 0 to 100 ps. Kinetics of the PIA and bleaching components were fitted with one and two exponentials, respectively (shown as the black-dashed lines in the inset). **d** Kinetics of the bleaching component measured at 80 K using 500-nm pump under various excitation carrier densities. Inset shows the kinetics from 0 to 100 ps. **e** Kinetics of the bleaching component measured at different temperatures using 500-nm pump under a fixed excitation carrier density of 35 × 10^18^ cm^−3^. **f** kinetics of the PIA component measured at 80 K using 500-nm pump under various excitation carrier densities. Inset shows the kinetics from 0 to 100 ps

We excited the MAPbI_3_ film at 80 K using 500-nm above-bandgap pumping and monitored the N–H stretching modes over a 3.5-ns time window. To achieve appreciable lattice temperature rise and drive the sub-lattices into non-equilibrium, we used pump fluences higher than those typically employed in transient absorption measurements that probe the electronic responses^13,15,27^. The excitation carrier density (denoted as *n*_0_) induced by the employed pump fluence is in the range of 4.5 × 10^18^ cm^−3^ to 93 × 10^18^ cm^−3^ (~1000–10,000 times of that can be reached under 1.5 AM condition; also see Supplementary Fig. 4 and Supplementary Note 2), which is comparable to those used in the studies of transient vibrational response^28^ or Auger heating^29^ in MAPbI_3_. The acquired ΔOD transient spectral map (Fig. 2a; here ΔOD denotes the transient change in absorbance) exhibits a short-lived (~10–20 ps), broadband photoinduced absorption (PIA), followed by a much longer-lived, narrow bleaching feature that grows in amplitude with time and persists over the measured time window up to 3.5 ns. Global analysis of the transient spectral map (see Supplementary Fig. 5 for the accuracy of the global analysis) conveys two principle components (PCs) respectively dominated by PIA and bleaching, and permits examination of their spectra (Fig. 2b) and dynamics (Fig. 2c). In Fig. 2b, the bleaching dips spectrally match the statically observed N–H modes (mode-I and mode-II), whereas the PIA component exhibits a derivative-like feature around the N–H modes (i.e., a dip on the red side and a peak on the blue side of the N–H mode). The derivative-like PIA features around the N–H modes cannot be assigned to excited-state absorption of the N–H-stretching modes, because anharmonicity of the atomic vibrational modes should yield excited-state absorption (i.e., a peak) on the red side of the statically measured ground state absorption of the N–H vibrational modes^30^. Instead, the broadband PIA, which manifests a positive change of the imaginary permittivity, induces a corresponding change of the real permittivity through the Kramers–Kronig relation, which in turn produces the spectrally localized, derivative-like PIA features around the ground-state absorption of the N–H modes (see Supplementary Figs. 6 and 7 and Supplementary Note 3).

The dynamics shown in Fig. 2c from global analysis demonstrates drastically different temporal characteristics of the PIA and bleaching components. While both the PIA and bleaching components exhibit a similarly fast, 5-ps exponentially-fitted timescale (Fig. 2c inset), at ~20 ps the bleaching component only reaches ~60% of its maximal amplitude, in comparison to the PIA component that has nearly completely decayed to zero. Time-resolved PL measurements with a streak camera (Supplementary Figs. 8–14) show that, under the range of *n*_0_ adopted in our TA experiments, amplified spontaneous emission (ASE) dominates the emission especially for the high *n*_0_ regime^31^. Based on the fact that the ASE exhibits an instrument-response-time-limited, sub-20-ps decay time (Supplementary Fig. 10), which is consistent with the fast decay time of the PIA component (Fig. 2c, inset), we can attribute the PIA to intra-conduction-band and intra-valence-band absorption by photo-excited carriers. The assignment of PIA in the infrared regime to carrier-excited-state absorption is consistent with a recent literature report^32^. On the other hand, the bleaching component resembles the static differential absorbance (Fig. 1d) and hence implies heating of the lattice with time. Here we attribute the fast timescale of the bleaching component to the excitation of low-energy phonon modes (denoted as LEPMs), including various optical phonon modes (via Fröhlich coupling) and acoustic phonon modes (through deformation potential and thermoelastic effects), due to hot carrier relaxation^14,27,33^. Population of these LEPMs induces thermalization of the hydrogen bond network, which weakens the hydrogen bonds and with it the oscillator strength of the N–H vibrational modes. Recent time-resolved electron scattering experiments demonstrate the excitation of low-frequency motions of the Pb-I cages on a ~10-ps timescale following above-bandgap photoexcitation^28^. Note that the LEPMs include modes of the heavy inorganic sub-lattice as well as low-frequency modes^8^ of the organic sub-lattice. Earlier study suggests that near-equilibrium electron-phonon scattering is dominated by longitudinal optical phonons involving primarily the inorganic sub-lattice^14^; whether the organic LEPMs can be directly scattered off (and hence excited) by hot carriers under the high excitation fluence regime used in this work is a subject that warrants further study^34^. We also note that, under the high *n*_0_ used in this work, Auger recombination and the accompanying Auger heating should take place (Supplementary Fig. 15). The Auger recombination rate, which has a third-order dependence on the instantaneous carrier density, is expected to drop at a similar rate as the fast decay of the PIA component (the latter is in turn dictated by the rate of ASE). As a result, besides hot carrier relaxation, we expect the fast rise of the bleaching component accompanying the fast decay of the PIA component to be also contributed by Auger heating. Lack of an apparent time lag between the fast decay of PIA and the fast rise of bleaching components is consistent with the sub-picosecond carrier thermalization timescale as reported elsewhere^35^.

In addition to the fast rise, the bleaching component further exhibits a much slower rise with a 220-ps exponentially-fitted timescale (Fig. 2c inset), suggesting an especially slow thermal process. The slow rise of the bleaching component cannot be attributed to heat diffusion away from the pumped region, which would otherwise lead to a decay rather than a rise. Note that, calculations of the carrier recombination processes using literature reported recombination rate constants^36^ show that Auger heating is completed within tens of picoseconds (Supplementary Fig. 16 and Supplementary Note 4), and hence cannot explain the much longer rise time of the bleaching component. Furthermore, the slow rise of the bleaching cannot be attributed the thermalization of the whole lattice, as such picture is not supported by the near-bandgap emission observed immediately following the above-bandgap pump excitation (Supplementary Figs. 8–14). Here, we attribute this slow rise of the bleaching component to the slow buildup of high-energy phonon modes (denoted as HEPMs) specific to the organic sub-lattice through phonon–phonon interactions between the LEPMs and HEPMs. To investigate this further, we performed fluence and temperature dependent measurements (Supplementary Figs. 17, 18). Figure 2d and Supplementary Fig. 19a present fluence-dependent dynamics and spectra of the bleaching component with 500-nm excitation at 80 K. We found the timescale of the bleaching to be largely fluence, or *n*_0_ independent (consistent with our theoretical calculation shown later), hence the slow component, as expected, is decoupled from charge carrier recombination. The bleaching dynamics measured at various sample temperatures exhibits a nearly negligible temperature dependence (Fig. 2e), and TA experiments using different above-bandgap pump wavelengths yield the same timescale of the bleaching component (not shown). In addition, measurements on an MAPbI_3_ film fabricated by a different method (Supplementary Figs. 20, 21) reveal similarly slow bleaching dynamics, confirming that the observed slow heating of lattice does not depend on the explored synthetic conditions but is intrinsic to the composition. The fluence-dependent dynamics and spectra of the PIA component are shown in Fig. 2f and Supplementary Fig. 19b, respectively. Although the fast decay of the PIA component exhibits fluence-independent timescale (Fig. 2f, inset), consistent with the fluence-independent ASE dynamics, we find that at low *n*_0_ regime (*n*_0_ < 19 × 10^18^ cm^−3^) the PIA decay further exhibits a slower timescale, which is attributed to second- and first-order carrier recombination.

### Two-temperature model

**Fig. 3:**
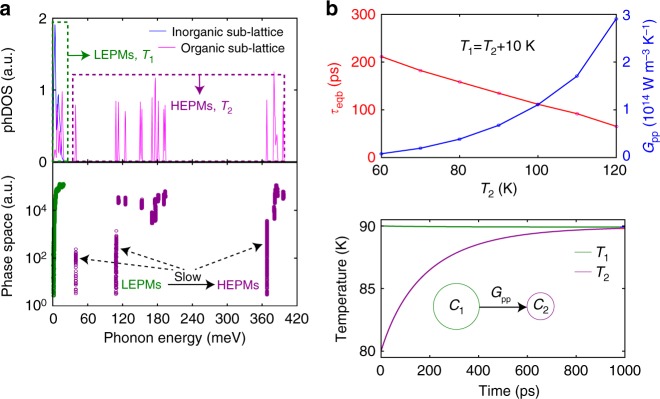
Theoretical calculation of phonon–phonon coupling and the two-temperature model. **a** Projected phonon density of states (phDOS) of pseudo-cubic MAPbI_3_ and phase space for phonon emission, both plotted as functions of energy and are shown in arbitrary units. Low-energy phonon modes (LEPMs) (<20 meV) and high-energy phonon modes (HEPMs) (>20 meV) are highlighted. **b** Top: calculated dependences of *τ*_eqb_ and *G*_pp_ on *T*_2_ (varied from 60 K to 120 K in increments of 10 K) with a fixed Δ*T* of 10 K. Bottom: representative temperature evolution of the two phonon subsets with an initial *T*_2_ of 80 K and *T*_1_ of 90 K

To understand the slow phonon equilibration, we developed a two-temperature model (TTM) where the LEPMs (energy <20 meV) and HEPMs (energy >20 meV) assume effective temperatures of *T*_1_ and *T*_2_, respectively (Fig. 3a). The 20-meV cut-off energy used in our TTM calculation corresponds to the upper bound of the phonon frequencies of the inorganic sub-lattice as well as the low-frequency modes of the organic sub-lattice. We found that calculation with slight variation of the cut-off energy does not alter the conclusion of the calculation. Energy transfer between the two subsets of phonon modes is described by $\frac{{{\mathrm d}T_1}}{{{\mathrm d}t}} = \frac{{G_{{\rm pp}}}}{{C_1}}\left( {T_2 - T_1} \right)$ and $\frac{{{\mathrm d}T_2}}{{{\mathrm d}t}} = - \frac{{G_{{\rm pp}}}}{{C_2}}\left( {T_2 - T_1} \right)$, where *G*_pp_, computed from first-principles, represents an effective coupling coefficient between the two phonon subsets. Solution of the above equations results in $\left( {T_2 - T_1} \right)\sim {\mathrm{exp}}\left[ { - \left( {\frac{{G_{{\rm pp}}}}{{C_1}} + \frac{{G_{{\rm pp}}}}{{C_2}}} \right)t} \right]$. Since the heat capacity of the LEPMs (*C*_1_) greatly exceeds that of the HEPMs (*C*_2_), $\frac{{C_2}}{{G_{{\rm pp}}}}$ represents the effective timescale of thermal equilibration. The large spectral mismatch between the HEPMs of the organic sub-lattice and the LEPMs has important consequences on the phonon–phonon interactions due to the requirement of energy and momentum conservation: the phase space for phonon emission (Fig. 3a) shows that many of the HEPMs have a scattering phase space that is one to three orders-of-magnitude smaller than that of the LEPMs, which, as shown in Fig. 3b, results in *G*_pp_ of 10^13^ to 10^14^ W m^−3^ K^−1^ over a temperature range of 60 to 120 K, that is much smaller than typical values^37^ on the order of 10^16^–10^17^ W m^−3^ K^−1^. With the computed *G*_pp_, we calculated the dependence of equilibration time-constant (*τ*_eqb_) on *T*_2_ with a fixed value of Δ*T* (Fig. 3b), and furthermore the time evolution of *T*_1_ and *T*_2_ starting with *T*_2_ = 80 K and *T*_1_ = *T*_2_ + Δ*T* = 90 K (Fig. 3b) assuming a Δ*T* of 10 K arising from energy transfer from the photoexcited carriers to the LEPMs. A long timescale of equilibration (~230 ps exponentially-fitted time constant) is obtained, which is comparable to the experimental results. Calculations also show negligible changes (within 1%) of *G*_pp_ with Δ*T* varying from 5 K to 20 K, consistent with the fluence independence of the bleaching dynamics shown in Fig. 2d, and suggests that non-equilibrium between the LEPMs and HEPMs can be relevant even at low excitation fluences. While the physics of the scattering phase-space-limited energy transfer between LEPMs and HEPMs is elucidated through the TTM, the predicted *τ*_eqb_ exhibits a larger temperature dependence (70 – 220 ps for *T*_2_ ranging from 60 K to 120 K with a fixed Δ*T* of 10 K) in comparison to the measured bleaching kinetics (Fig. 2e). The present calculations of vibrational spectrum and 3rd-order force constants (that determine energy transfer between the phonon modes) were performed on a symmetric cubic structure at 0 K. However, increase in temperature results in stronger atomic thermal motions as well as octahedra-tilting, which are expected to screen the vibrational coupling between the inorganic and organic sub-lattices through reduction in the effective hydrogen bond strength^38,39^. The incorporation of such temperature-resolved anharmonic force constants may provide better quantitative agreement of calculated equilibration times with experiments.

### Lattice heating inferred from transient absorption results

**Fig. 4:**
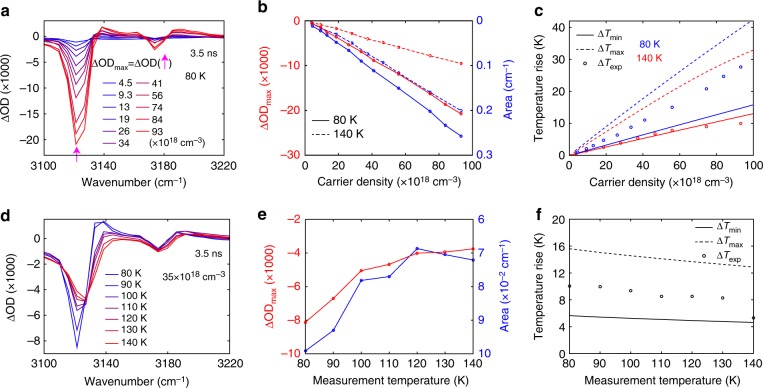
Transient lattice heating of MAPbI_3_. **a** Transient spectra at delay time of 3.5 ns measured at 80 K under various *n*_0_. **b** Red: *n*_0_ dependent ΔOD_max_ measured at 80 K (solid line) and 140 K (dashed line). Blue: *n*_0_ dependent area of Δ*OD* measured at 80 K (solid line) and 140 K (dashed line). Both ΔOD_max_ and area of ΔOD are defined similarly to the static case as shown in Fig. 1d. **c** Circles: lattice temperature rise deduced by comparing the areas of ΔOD obtained from static (Fig. 1d) and transient (Fig. 4b) measurements. Solid lines: estimated minimal temperature rise. Dashed lines: estimated maximal temperature rise. **d** Transient spectra at delay time of 3.5 ns measured at different temperatures under fixed *n*_0_ of 35 × 10^18^ cm^−3^. **e** ΔOD_max_ (red) and area of ΔOD (blue) obtained from transient measurements at different temperatures under a fixed *n*_0_ of 35 × 10^18^ cm^−3^. **f** Lattice temperature rise deduced by comparing the areas of ΔOD obtained from static and transient measurements performed at different temperatures under a fixed *n*_0_ of 35 × 10^18^ cm^−3^. Solid line and dashed line indicate respectively the estimated minimal and maximal temperature rises similar to those shown in **c**

As demonstrated in Fig. 1d, the static lattice temperature rise can be effectively characterized by the area of ΔOD of mode-I, which we define as the product of ΔOD_max_ (the highest negative value of ΔOD of mode-I; Fig. 1d inset) and the corresponding full-width-half-maximum. Figure 2d, e show that the rise of the bleaching component is nearly complete at 3.5-ns delay time, which is the longest delay time of our setup. Therefore, comparing the statically measured area of ΔOD with the transient analogue captured at 3.5-ns delay time, when the sample is at nearly thermal equilibrium, can inform on the pump-induced lattice temperature rise. Figure 4a and Supplementary Fig. 19c present the *n*_0_ dependent transient ΔOD spectra at 3.5-ns delay time measured at 80 K and 140 K, respectively. The determined ΔOD_max_ and area of ΔOD are plotted in Fig. 4b against *n*_0_. Comparison of Fig. 4b with the inset of Fig. 1d yields Fig. 4c, which presents the *n*_0_ dependent lattice temperature rise. We also calculated the upper or lower bounds on the lattice temperature rise, by assuming that each photon contributes, respectively, energy of *ħω* or (*ħω*-*E*_g_) to lattice heat. The temperature dependent lattice heat capacity is taken from an earlier literature report^40^. We found that, for measurements performed at 80 K, the estimated temperature rise achieved in TA experiments at the low *n*_0_ regime lies near the lower bound, but under higher *n*_0_ it approaches the average of the lower and upper bounds. This observation indicates a higher efficiency of Auger heating achieved at higher *n*_0_. For measurements performed at 140 K (which is ~10 K below the tetragonal-to-orthorhombic phase transition), the deduced lattice temperature rise is near the lower bound throughout the entire explored range of *n*_0_. Time-resolved PL measurements (Supplementary Fig. 11) reveal the preservation of orthorhombic phase measured at 140 K under very high fluence (400 × 10^18^ cm^−3^) during the time window of the streak camera measurement, evident from its qualitatively different PL spectra from that taken at 150 K in the tetragonal phase (Supplementary Fig. 13). Transient spectra at 3.5-ns delay time measured at different sample temperatures under a fixed *n*_0_ is presented in Fig. 4d. The ΔOD_max_ and area of ΔOD, and the deduced temperature rise are plotted in Fig. 4e and f, respectively. We found that the lattice temperature rise decreases with an increasing measurement temperature, which stems from the temperature dependent heat capacity^40^.

### Static and transient studies of FAPbI_3_

**Fig. 5:**
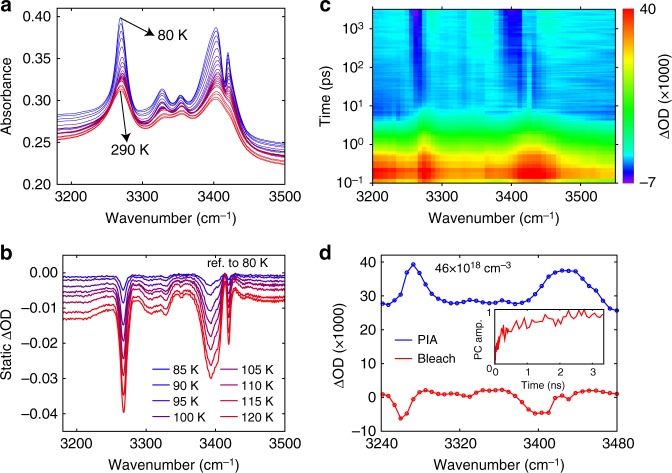
Static and transient absorption measurements of FAPbI_3_. **a** Static temperature dependent absorbance from 80 K to 290 K in increments of 10 K. **b** Differential absorption spectra of the N–H stretching modes in the range of 3200 cm^−1^ to 3500 cm^−1^. **c** Transient spectral map taken at 80 K using 500 nm pump excitation (329 µJ cm^−2^ fluence). The color-coded quantity is ΔOD (×1000). **d** Spectra of the two principle components (PCs) of the transient response shown in red (bleaching) and blue (photo-induced absorption, or PIA). Circles correspond to the pixels of the array detector. Inset: kinetics of the bleach principle component

To demonstrate that slow thermal equilibration is a common feature of HOIPs, we further examined FAPbI_3_, which also enables exceptional solar-cell efficiency^41^. The static spectra (Fig. 5a) demonstrate strong absorption features in the range of 3200 cm^−1^ to 3500 cm^−1^ attributable to the N–H stretching modes^42^, which decrease in intensity upon heating. The differential absorption spectra (referenced to 80 K) measured with FTIR is shown in Fig. 5b. A transient spectral map (Fig. 5c) acquired using 500 nm excitation (329 µJ cm^−2^ fluence) reveals a bleaching feature indicative of heating of the lattice. Global analysis of the transient spectral map reveals both a PIA and a bleaching principle component, with the latter exhibiting a slow rise time (Fig. 5d, inset) similar to the case of MAPbI_3_. Static and transient measurements of the C = N stretching modes at around 1700 cm^−1^ also show a slow timescale (Supplementary Figs. 22, 23, 24 and Supplementary Note 5). We note that transient measurements on MAPbI_3_ were limited to the orthorhombic phase with rotations of MA^+^ suppressed. Measurements of the tetragonal phase of MAPbI_3_ are hindered by the large mismatch of its vibrational and electronic absorption cross sections, and a weaker temperature dependence of vibrational absorption (see Supplementary Fig. 3). For FAPbI_3_, the free rotations of FA^+^ are retained at the temperature of measurements^43^. The slow thermal equilibration observed for FAPbI_3_ is consistent with the recent study on superatomic crystals^44^, which demonstrates that the rotational degree of freedom of the organic sub-lattice may further reduce the phonon–phonon coupling between the weakly-coupled organic and inorganic sub-lattices.

## Discussion

In summary, we investigated lattice thermalization process in HOIPs by leveraging their unique mid-infrared vibrational absorption, and demonstrate an unusually slow build-up of thermal equilibrium between the organic and inorganic sub-lattices up to a couple of nanoseconds. Our results suggest that classical temperature models and heat transport equations may not be suitable for describing HOIPs under large temperature gradients or impulsive lattice heating. The long-lasting thermal non-equilibrium in HOIPs may alter the electronic configuration and contribute to a low thermal conductivity^45,46^, which can be especially important in two-dimensional HOIPs^47^ wherein the organic and inorganic sub-lattices are connected in series. Our results also inform on optically induced, sequential lattice heating in HOIPs at intermediate and high excitation fluence regimes relevant for solar concentration, high brightness light-emitting diodes, and lasing applications. Design of device configurations with better impedance matching between the phonon modes of the inorganic sub-lattice and the nearby substrate may allow the extraction of heat out of the lattice before the population of various high-frequency organic modes, thereby contributing to an improved thermal stability of HOIPs. Although the demonstrated experimental approach cannot be used for the examination of lattice thermalization in all-inorganic perovskites (e.g., CsPbBr_3_) due to the lack of high-frequency vibrational modes in the mid-infrared range, we expect the slow thermal equilibration between the organic and inorganic sub-lattices is unique to HOIPs. Transiently monitoring the absorbance of organic sub-lattice-related vibrational modes as a probe of the lattice temperature demonstrated in this work can be generalized to the study of energy dissipation pathways in the chemically diverse HOIPs^48^, as well as polymeric and small-molecule semiconductors^49^. Such technique can also inform on the interactions between polarization and lattices in newly discovered HOIPs exhibiting large ferroelectric responses^50^, and thermal behaviors of other hybrid systems such as metal–organic frameworks^51^ and polyoxometalate–organic solids^52^. Transient absorption measurements of the temperature-sensitive, mid-infrared organic modes with electronically enabled microsecond delay time instead of the nanosecond delay time window used in this work, may further provide a way of characterizing the near-equilibrium heat transport properties of MAPbI_3_ and other hybrid materials.

## Methods

### Chemicals

Methylamine solution (40 wt.% CH_3_NH_2_ in H_2_O), formamidine acetate salt (HC(NH_2_)NH∙HCOOCH_3_ 99%), *N*-methyl-2-pyrrolidinone (NMP) and γ-butyrolactone (GBL) were purchased from Sigma-Aldrich. Hydriodic acid (57 wt.% HI in H_2_O), N,N-dimethylformamide (DMF, anhydrous, 99.8%) and lead(II) iodide (PbI_2_, 99.9985% metals basis) were purchased from Alfa Aesar. Ethyl ether (anhydrous) was purchased from Fisher Chemical. CH_3_NH_3_I was synthesized according to the method reported before^54^. Briefly, HI was dropwise introduced into equimolar of CH_3_NH_2_ in a round-bottom flask immersed in an ice bath, and the solution was then rotary-evaporated at 60 °C to dry off the water. The resulting yellow-colored solid was washed with ethyl ether on filter papers, accompanied by vacuum filtration. The washed CH_3_NH_3_I powder was then dried in a vacuum oven at 80 °C overnight. HC(NH_2_)_2_I was prepared by mixing HC(NH_2_)NH∙HCOOCH_3_ and HI in 1:1 molar ratio in a round-bottom flask immersed in ice bath without stirring. The round-bottom flask was then sealed and the reaction mixture was gently stirred for 6 h in ice bath. Next, the stirred mixture was rotary-evaporated at 80 °C to dry off the solvent (~95% of original solution volume), after which the evaporator water bath was gradually cooled to room temperature with appearance of white precipitates. Finally, the round-bottom flask containing the saturated solution with precipitate was transferred into a vacuum oven, and dried at 80°C overnight to generate white-colored powder. Pure form of HC(NH_2_)_2_I was obtained after being washed with ethyl ether, and dried in the vacuum oven again.

### MAPbI3 film fabrication

We prepared MAPbI_3_ thin film following previous methods^55,56^. Specifically, 50 wt.% precursor solution was obtained by dissolving 1.2:1 molar ratio of CH_3_NH_3_I:PbI_2_ in 7:3 weight ratio of NMP:GBL. The precursor solution was spin-coated on one-inch-diameter CaF_2_ wafer (Thorlabs) at 1300 rpm. The wet film on CaF_2_ was quickly soaked in diethyl ether for 300 s, after which the film was annealed at 150 °C for 20 min on a hot plate in a humidity controlled environment, covered by a petri dish. X-ray diffraction pattern and scanning electron micrograph of the fabricated film are shown in Supplementary Figs. 21 and 25. Time integrated and time-resolved PL spectra are shown in Supplementary Fig. 26. The obtained film has hundreds of ns lifetime at 295 K (measured using low-fluence, picosecond diode laser), indicating low defect density.

### FAPbI_3_ film fabrication

1:1 molar ratio of HC(NH_2_)_2_I and PbI_2_ was dissolved in GBL with 1 M concentration. Next, the solution was spin-coated onto CaF_2_ substrates at 1300 rpm for 25 s. Wet films were then quickly transferred into ethyl ether bath to develop for 180 s. Yellow-colored films were formed and dried under nitrogen flow, then annealed at 150 °C for 25 min on a hot plate with petri dishes covered atop in a humidity-controlled environment. Time-integrated and time-resolved PL spectra are shown in Supplementary Fig. 27.

### Structural and optical characterization

X-ray diffraction data was collected using a Bruker D2 Phaser Diffractometer. SEM images were captured using Hitachi SU8030. PL spectra and time-correlated-single-photon-counting were measured under 405 nm photoexcitation with 35-ps pulse-width laser diode. Emitted photons were detected with a CCD or avalanche photodiode, respectively. A streak camera was used to collect temporally and spectrally resolved PL data. Static infrared absorbance spectra were acquired using an FTIR (Thermo Nicolet 6700) with a spectral resolution of 0.5 cm^−1^. Additional temperature dependent FTIR data on MAPbI_3_ and MAI are presented in Supplementary Figs. 28 and 29. Visible-pump, infrared-probe experiments were performed using a titanium:sapphire amplifier with 2 kHz repetition rate and 35 fs pulse width. The visible pump pulses were generated by an optical parametric amplifier and were reduced to 1 kHz repetition rate. The mid-infrared probe pulses were produced by difference frequency mixing of signal and idler beams using a separate optical parametric amplifier (see Supplementary Fig. 30 for a representative probe spectrum). Details of the experimental setup have been described elsewhere^57^. Samples were mounted in a liquid-nitrogen-cooled, cold-finger cryostat with a base pressure below 1 × 10^−6^ Torr in all the optical measurements. Each of the transient spectral map was acquired by averaging over several (usually 2 to 4) independent time-delay scans, during which no change of transient spectra or kinetics was observed. Similarly, the PL spectra (shown in Supplementary Fig. 15) were obtained by measuring the same spot on the sample within the entire explored range of fluence over tens of minutes, with no change of sample property. Such sample integrity is attributed to the low sample temperature (within the orthorhombic phase) of the measurement.

### Density functional calculations

Vienna Ab initio simulation package (VASP) was used to perform density functional calculations^58,59^. Projector-augmented wave (PAW) method^60^ in conjunction with the Perdew-Burke-Ernzerhof revised for solids (PBEsol)^61,62^ within the generalized gradient approximation (GGA)^63^ for the exchange-correlation functional were used. A plane wave basis with a kinetic energy cutoff of 700 eV was used. The force and energy convergence thresholds were set to be 10^−3^ eV·Å^−1^ and 10^−8^ eV, respectively. The methylammonium ion (CH_3_NH_3_^+^) was sampled with *Γ* point only in a cubic box with edge-length of 20 Å. Structure of O-phase of MAPbI_3_ was obtained from reference^64^ and sampled by a 4 × 4 × 2 **k** point mesh. To partially incorporate temperature effect, we expanded the 0-K relaxed structure by 0.5% in lattice constant isotropically^25^. 1 × 1 × 1 supercell structures were constructed to extract interatomic force constant. Zone center phonon frequency and density of states were computed using the Phonopy package^65^.

### Two-temperature model calculations

The calculation of an effective phonon–phonon coupling coefficient *G*_pp_(*T*_1_, *T*_2_) involves the computation of total energy transfer between LEPMs (energy <20 meV) at temperature *T*_1_ and HEPMs (energy >20 meV) at temperature *T*_2_. The third order force constants required for phonon–phonon interactions were obtained using compressive sensing lattice^66^, during which we adopted a computationally manageable, pseudo-cubic crystal structure^64^ of MAPbI_3_. In the compressive sensing lattice dynamics, randomly perturbed atomic displacements (~0.01 A) were used to construct training set for interatomic force constants (IFCs) fitting (see Supplementary Fig. 31 for accuracy of fitted IFCs by comparing predicted forces with DFT forces). Although experiments were performed on the orthorhombic phase, the phDOS of the cubic and orthorhombic phase are qualitatively similar (Figs. 1b and 3a), and the weak scattering phase space for energy transfer between the two phonon subsets is expected to be present for both phases. The phase space for phonon emission (Fig. 3a) for a given vibrational mode $\left| {{\boldsymbol{q}}v} \right\rangle$ is given by

$P( {\omega _{{\boldsymbol{q}}v}} ) = \mathop {\sum }\limits_{{\boldsymbol{q}}\prime v\prime } \mathop {\sum }\limits_{v{\prime\hskip -1pt\prime} } \delta ( {\omega _{{\boldsymbol{q}}v} - \omega _{{\boldsymbol{q}}\prime v\prime } - \omega _{{\boldsymbol{q}}{\prime\hskip -1pt\prime} v{\prime\hskip -1pt\prime} }} )$, where ${\boldsymbol{q}}{\prime\hskip -1pt\prime} = {\boldsymbol{q}} - {\boldsymbol{q}}\prime + {\boldsymbol{G}}$, $v$ denotes the branch index and ***G*** denotes a reciprocal lattice vector^67^. To calculate *G*_pp_, we computed the net energy gained by a phonon mode $\left| {{\boldsymbol{q}}v} \right\rangle$ as $\Delta E_{{\boldsymbol{q}}v} = \hbar \omega _{{\boldsymbol{q}}v}\frac{{2\pi }}{{\hbar ^2}}\mathop {\sum }\limits_{{\boldsymbol{q}}\prime v\prime } \mathop {\sum }\limits_{v{\prime\hskip -1pt\prime} } \left\{ {\left| {{\mathrm{\psi }}_{{\boldsymbol{qq}}\prime {\boldsymbol{q}}_1^{\prime\hskip -.5pt\prime} }^{vv\prime v}} \right|^2\left[ {\left( {n_{{\boldsymbol{q}}v} + 1} \right)\left( {n_{{\boldsymbol{q}}\prime v\prime } + 1} \right)n_{{\boldsymbol{q}}_1^{\prime\hskip -.5pt\prime} v{\prime\hskip -1pt\prime} } - n_{{\boldsymbol{q}}v}n_{{\boldsymbol{q}}\prime v\prime }\left( {n_{{\boldsymbol{q}}_1^{\prime\hskip -.5pt\prime} v{\prime\hskip -1pt\prime} } + 1} \right)} \right] \delta \left( {\omega _{{\boldsymbol{q}}v} + \omega _{{\boldsymbol{q}}\prime v\prime } - \omega _{{\boldsymbol{q}}_1^{\prime\hskip -.5pt\prime} v{\prime\hskip -1pt\prime} }} \right) + \frac{1}{2}\left| {{\mathrm{\psi }}_{{\boldsymbol{qq}}\prime {\boldsymbol{q}}_1^{\prime\hskip -.5pt\prime} }^{vv\prime v{\prime\hskip -1pt\prime} }} \right|^2\left[ {\left( {n_{{\boldsymbol{q}}v} + 1} \right)n_{{\boldsymbol{q}}\prime v\prime }n_{{\boldsymbol{q}}_2^{\prime\hskip -.5pt\prime} v{\prime\hskip -1pt\prime} } - n_{{\boldsymbol{q}}v}\left( {n_{{\boldsymbol{q}}\prime v\prime } + 1} \right)\left( {n_{{\boldsymbol{q}}_2^{\prime\hskip -.5pt\prime} v{\prime\hskip -1pt\prime} } + 1} \right)} \right]\delta \left( {\omega _{{\boldsymbol{q}}v} - \omega _{{\boldsymbol{q}}\prime v\prime } - \omega _{{\boldsymbol{q}}_2^{\prime\hskip -.5pt\prime} v{\prime\hskip -1pt\prime} }} \right)} \right\}$ where ${\boldsymbol{q}}_1^{{\prime\hskip -.5pt\prime}} = {\boldsymbol{q}} + {\boldsymbol{q}}\prime + {\boldsymbol{G}}$, ${\boldsymbol{q}}_2^{{\prime\hskip -.5pt\prime}} = {\boldsymbol{q}} - {\boldsymbol{q}}\prime + {\boldsymbol{G}}$, Ψ denotes the three-phonon scattering matrix element, and $n_{{\boldsymbol{q}}v}$ denotes the occupation of mode $\left| {{\boldsymbol{q}}v} \right\rangle$. Here $n_{{\boldsymbol{q}}v}$ is calculated from the Bose–Einstein distribution function, which was evaluated at temperature *T*_1_ for the LEPMs and at temperature *T*_2_ for the HEPMs. The total energy transfer Δ*E*_1−2_ between the two subsets of phonons was obtained by summing over all phonon modes within a particular subset (the total energy gained/lost by the LEPMs equal the total energy lost/gained by the HEPMs). The phonon–phonon coupling coefficient *G*_pp_ is then given by Δ*E*_1−2_/(*T*_1_ − *T*_2_). For a given temperature *T*_2_, we set *T*_1_ higher than *T*_2_ by Δ*T* in evaluating *G*_pp_ for results in Fig. 3b. We found that *G*_pp_ is not sensitive to Δ*T* for the explored range from 5 K to 20 K. We used a Brillouin zone sampling of 16 × 16 × 16 and a smearing of 0.006 meV in the calculations of *G*_pp_.

### Data availability

All relevant data used in the article are available from the authors.

## Electronic supplementary material

Supplementary Infomation
