# Supplementary material for: Slow thermal equilibration in methylammonium lead iodide revealed by transient mid-infrared spectroscopy
Source: Nat Commun. 2018 Jul 18;9:2792. doi: 10.1038/s41467-018-05015-9 (PMC6052157; doi:10.1038/s41467-018-05015-9)
Supplement: Supplementary file 1 — Supplementary Infomation [file 41467_2018_5015_MOESM1_ESM.pdf]

Slow thermal equilibration in methylammonium lead iodide revealed by transient mid-infrared spectroscopy

P. Guo et al.

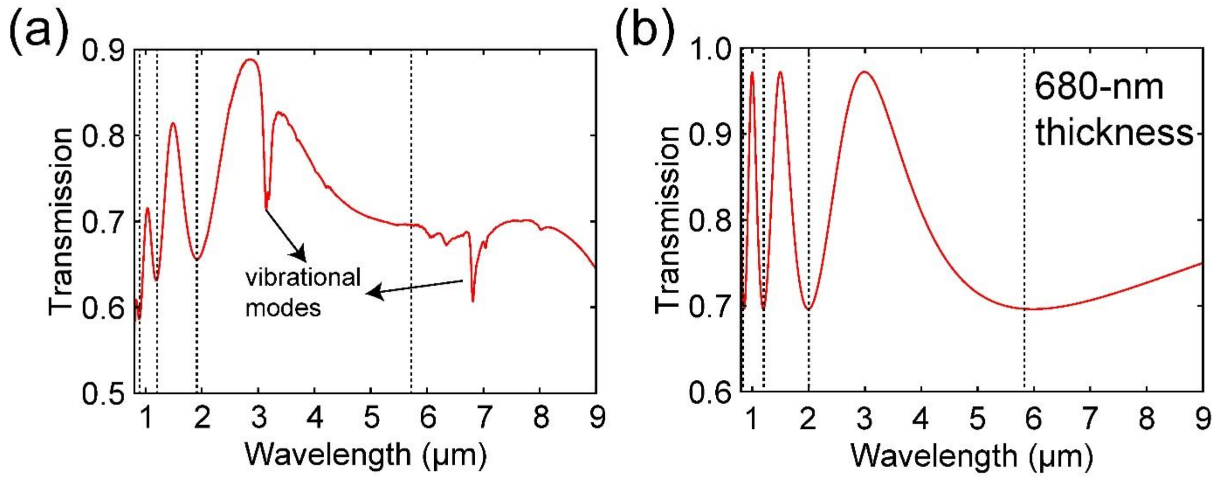

Supplementary Figure 1. (a) Measured transmission spectrum of the 680-nm thick $MAPbI_3$ film from 0.8 μm to 9 μm at 295 K. (b) Calculated transmission spectrum of a 680-nm thick $MAPbI_3$ film on CaF_2 substrate using the transfer-matrix method. The $MAPbI_3$ and CaF_2 assume Re(ε) of 4.85 and 2.04, respectively (see references[1,2]), and Im(ε) of 0. Dashed lines denote the Fabry-Perot-type transmission dips.

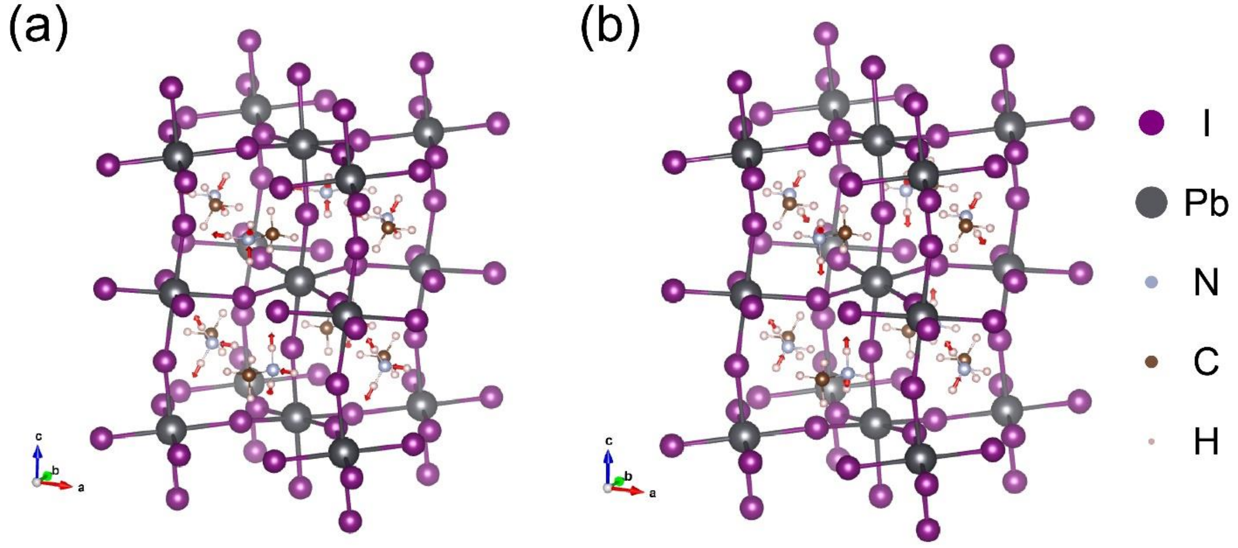

Supplementary Figure 2. First-principles calculated displacement vectors (red arrows) for the two asymmetric N-H stretching modes of orthorhombic $MAPbI_3$ with strong infrared absorbance. (a) Mode-I at 3107 cm^{-1}. (b) Mode-II at 3119 cm^{-1}.

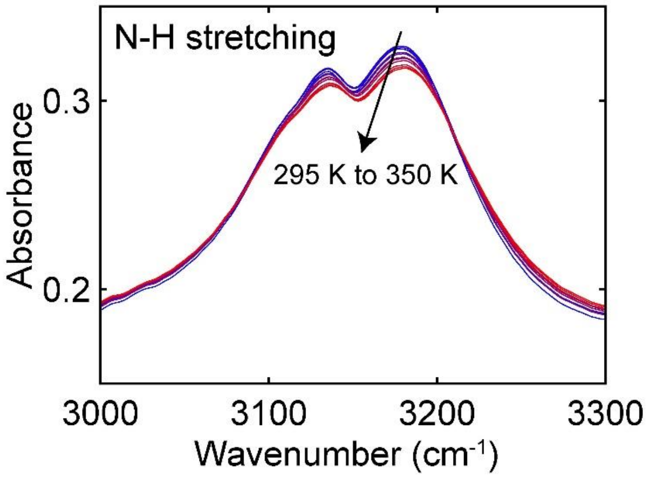

Supplementary Figure 3. Measured absorption spectra of the two asymmetric N-H stretching modes of $MAPbI_3$ from 295 K to 350 K in increments of 5 K. No discontinuous change of the absorbance is observed at the tetragonal-to-cubic phase transition temperature of ~327.4 K. Results shown in this figure were taken on a different sample from the one used in the TA experiments, to avoid possible sample degradation at high temperature.

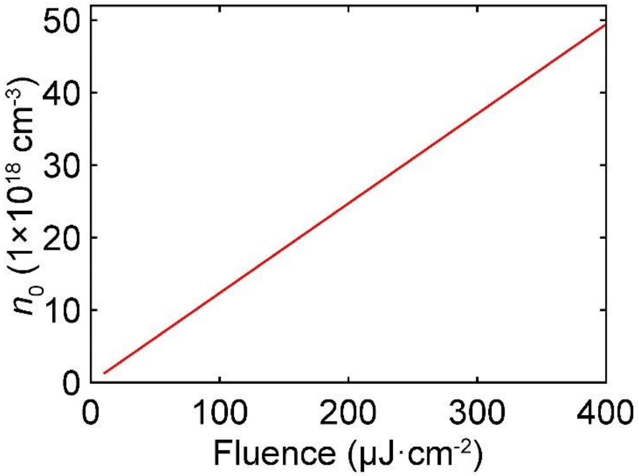

Supplementary Figure 4. Dependence of the excitation carrier density (n_0) on pump fluence.

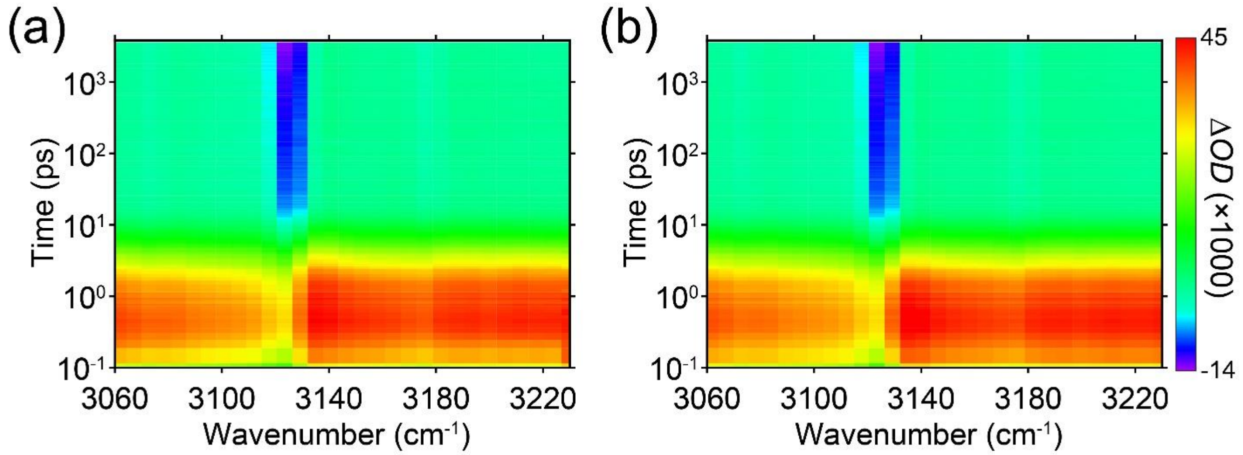

Supplementary Figure 5. (a) Experimentally measured Δ*OD* spectral map for $MAPbI_3$ at 80 K with 500-nm excitation (n_0=46×10^{18} cm^{-3}). (b) Δ*OD* spectral map reconstructed from the two principle components (PIA and bleaching) obtained from a global analysis of (a).

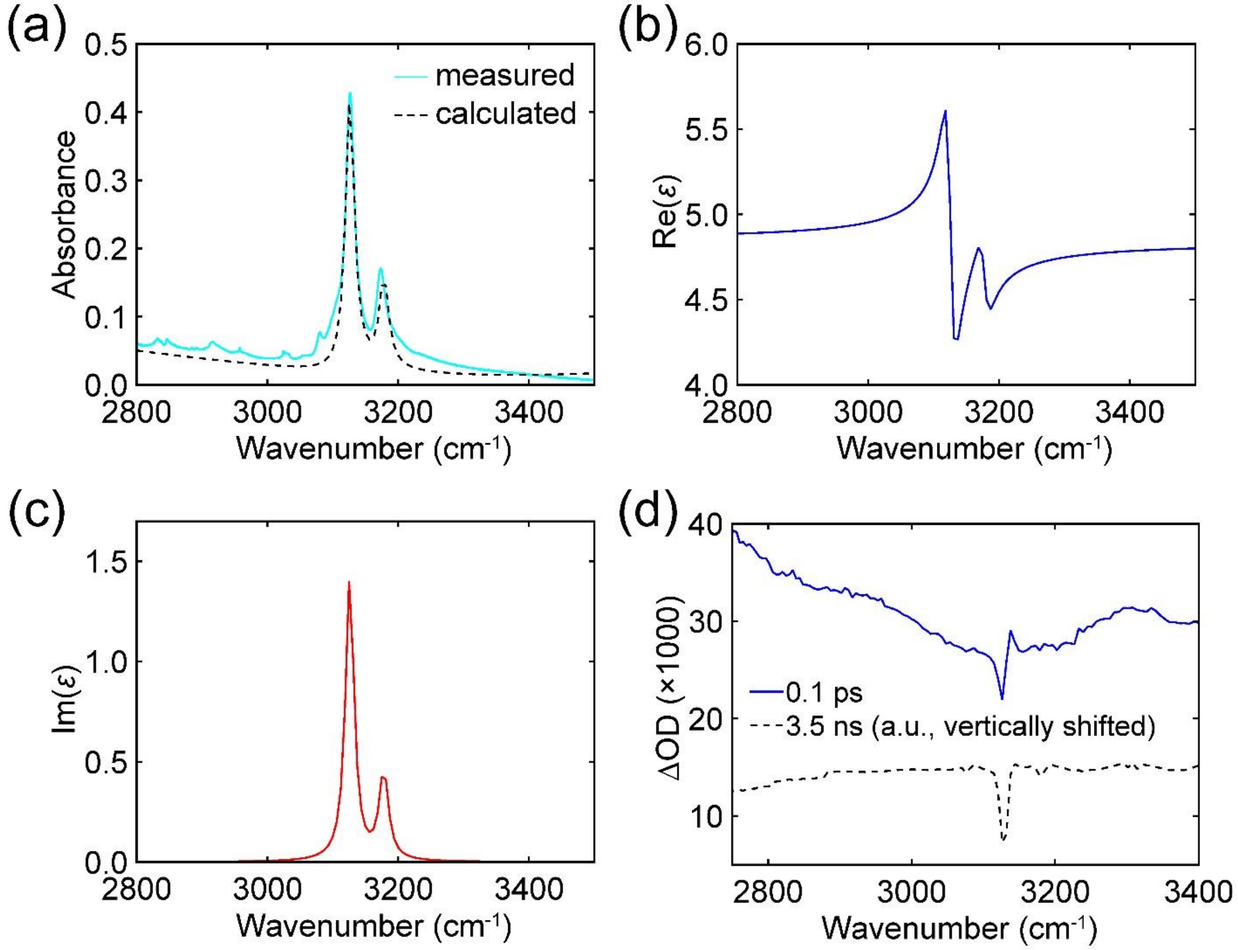

Supplementary Figure 6. (a) Measured (with FTIR, cyan line) and calculated absorbance (black-dashed line) of the $MAPbI_3$ film around the asymmetric N-H-stretching modes at 80 K. The measured absorbance has been subtracted by 0.1 from data shown in Fig. 1 to correct for the attenuation by the CaF_2 windows of the cryostat. (b) Fitted Re(ε) and (c) Im(ε) of the $MAPbI_3$ film around the N-H-stretching modes at 80 K. (d) Representative transient spectra captured at 0.1-ps and 3.5-ns delay times (n_0=28×10^{18} cm^{-3}).

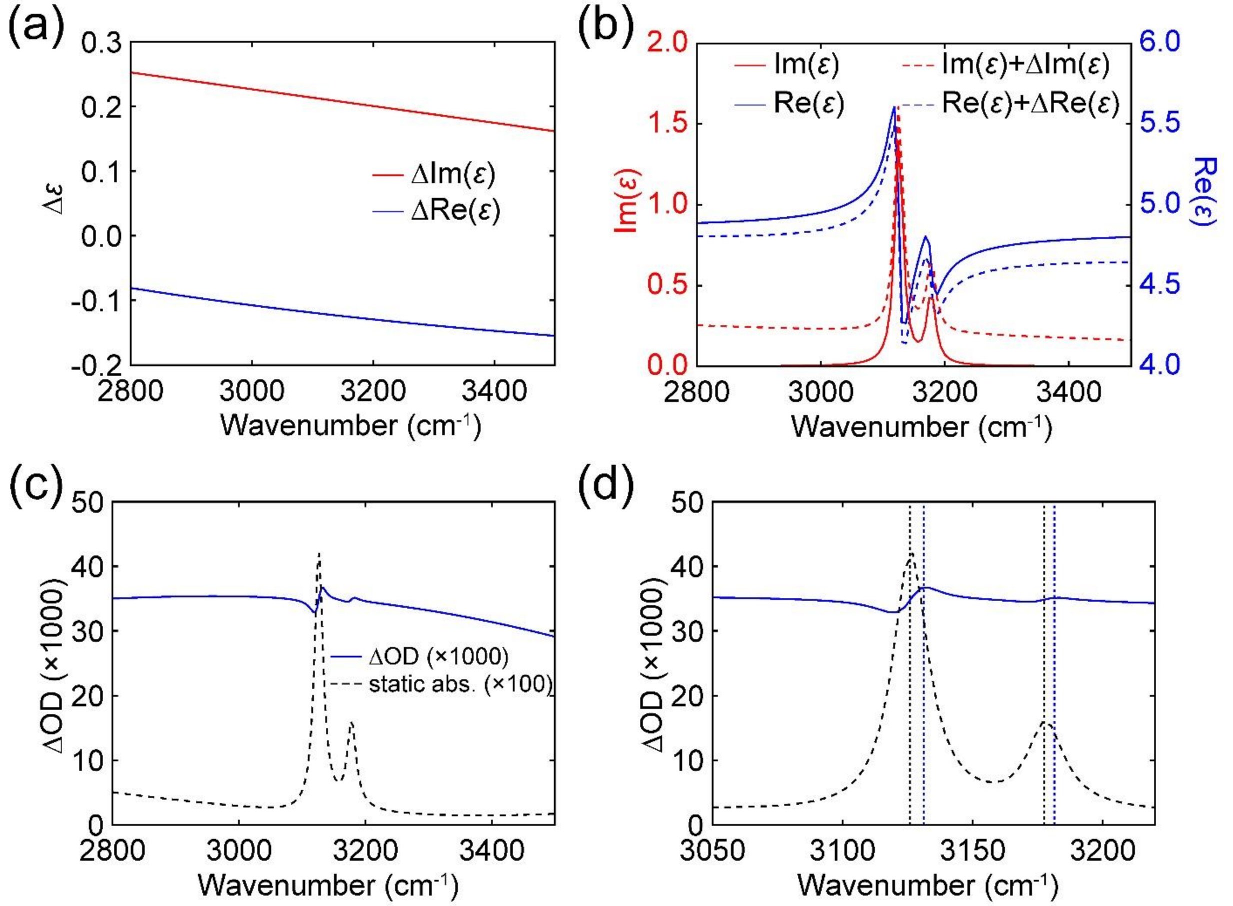

Supplementary Figure 7. (a) Arbitrarily introduced ΔIm(ε), and the corresponding ΔRe(ε) calculated from the Kramers-Kronig relationship. (b) Re(ε) and Im(ε) before (solid lines) and after the photo-induced changes (dashed lines). (c) The blue line shows the calculated change of absorbance due to ΔIm(ε) and ΔRe(ε); the black-dashed line shows the static absorbance. The calculated static absorbance is the same as that shown in Supplementary Fig. 6a (here magnified by 100 times). (d) Same as the data shown in (c) but plotted for the range of 3050 cm^{-1} to 3220 cm^{-1}.

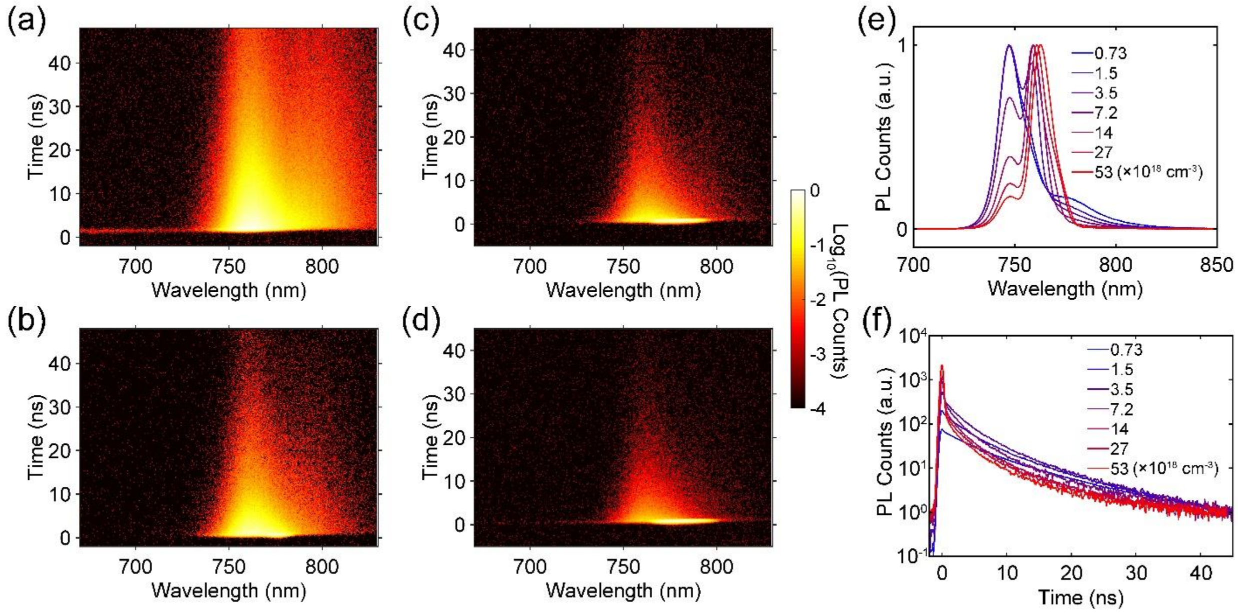

Supplementary Figure 8. (a) to (d) Time-resolved PL spectra (0~50 ns) for $MAPbI_3$ measured with a streak camera at 80 K under different n_0. (a) 0.73×10^{18} cm^{-3}; (b) 3.5×10^{18} cm^{-3}; (c) 14×10^{18} cm^{-3}; (d) 53×10^{18} cm^{-3}. The data shown in (a) to (d) are plotted under $\log_{10}$ scale with the corresponding scalebar shown on the right. (e) Time-integrated PL spectra measured with a CCD camera under different n_0. (f) Dynamics of the PL (0~50 ns) under different n_0, integrated over the entire spectral window as shown in (a) to (d).

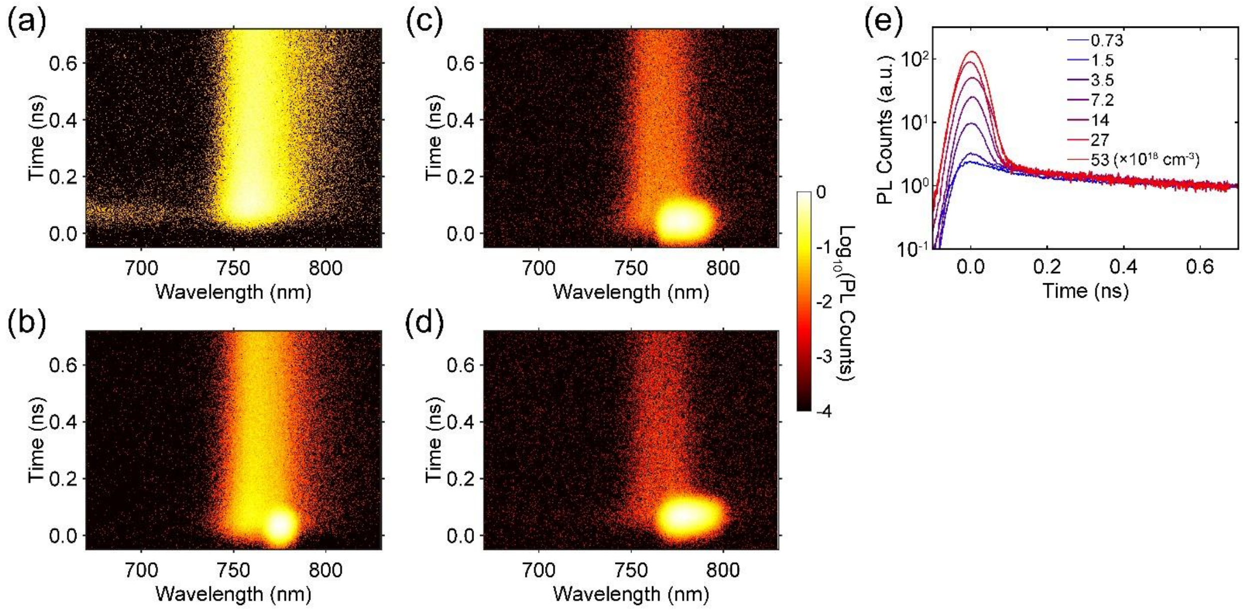

Supplementary Figure 9. (a) to (d) Time-resolved PL spectra (0~0.7 ns) for $MAPbI_3$ measured with a streak camera at 80 K under different n_0. (a) 0.73×10^{18} cm^{-3}; (b) 3.5×10^{18} cm^{-3}; (c) 14×10^{18} cm^{-3}; (d) 53×10^{18} cm^{-3}. The data shown in (a) to (d) are plotted under log_{10} scale with the corresponding scalebar shown on the right. (e) Dynamics of the PL (0~0.7 ns) under different n_0, integrated over the entire spectral window as shown in (a) to (d).

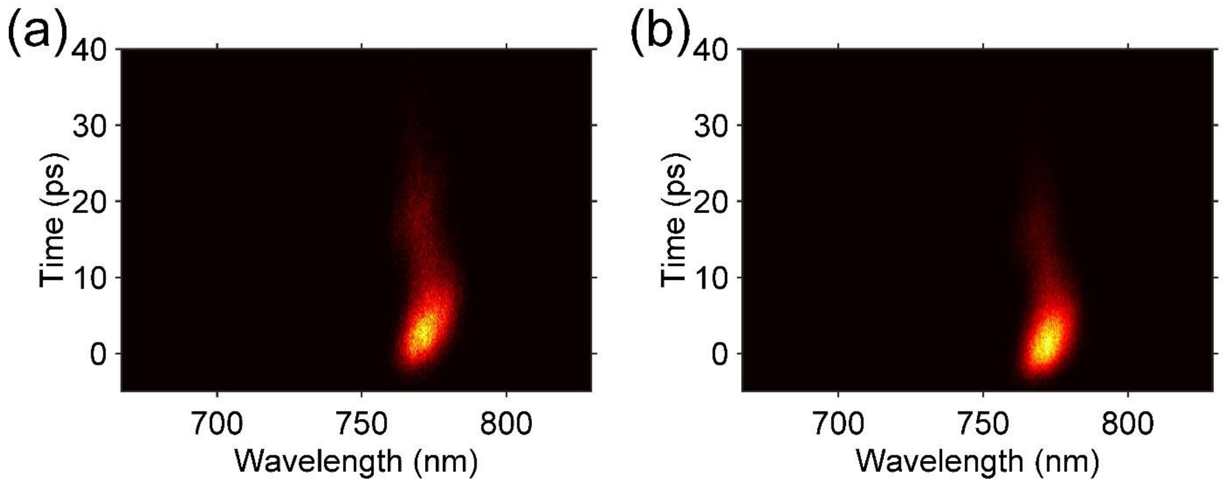

Supplementary Figure 10. Time-resolved PL spectra (0~40 ps) for $MAPbI_3$ measured with a streak camera at 80 K under different n_0. (a) 4.3×10^{18} cm^{-3}; (b) 10.5×10^{18} cm^{-3}.

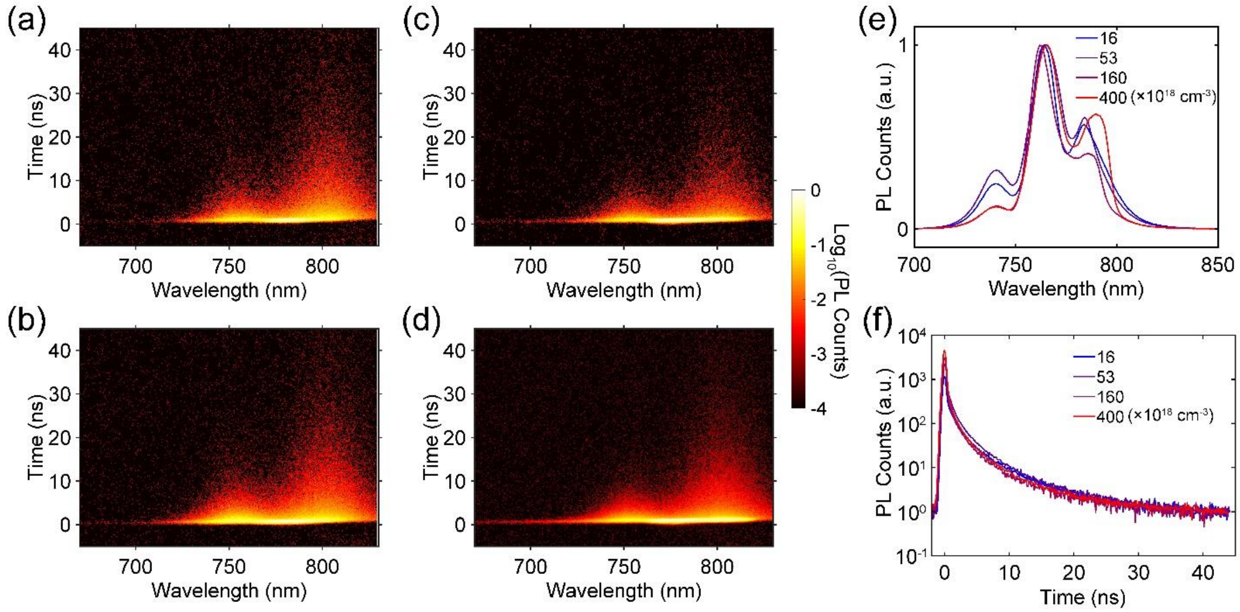

Supplementary Figure 11. (a) to (d) Time-resolved PL spectra (0~50 ns) for $MAPbI_3$ measured with a streak camera at 140 K under different n_0. (a) 16×10^{18} cm^{-3}; (b) 53×10^{18} cm^{-3}; (c) 160×10^{18} cm^{-3}; (d) 400×10^{18} cm^{-3}. Data shown in (a) to (d) are plotted under $\log_{10}$ scale with the corresponding scalebar shown on the right. (e) Time-integrated PL spectra measured with a CCD camera under different n_0. (f) Dynamics of the PL (0~50 ns) under different n_0, integrated over the entire spectral window shown in (a) to (d).

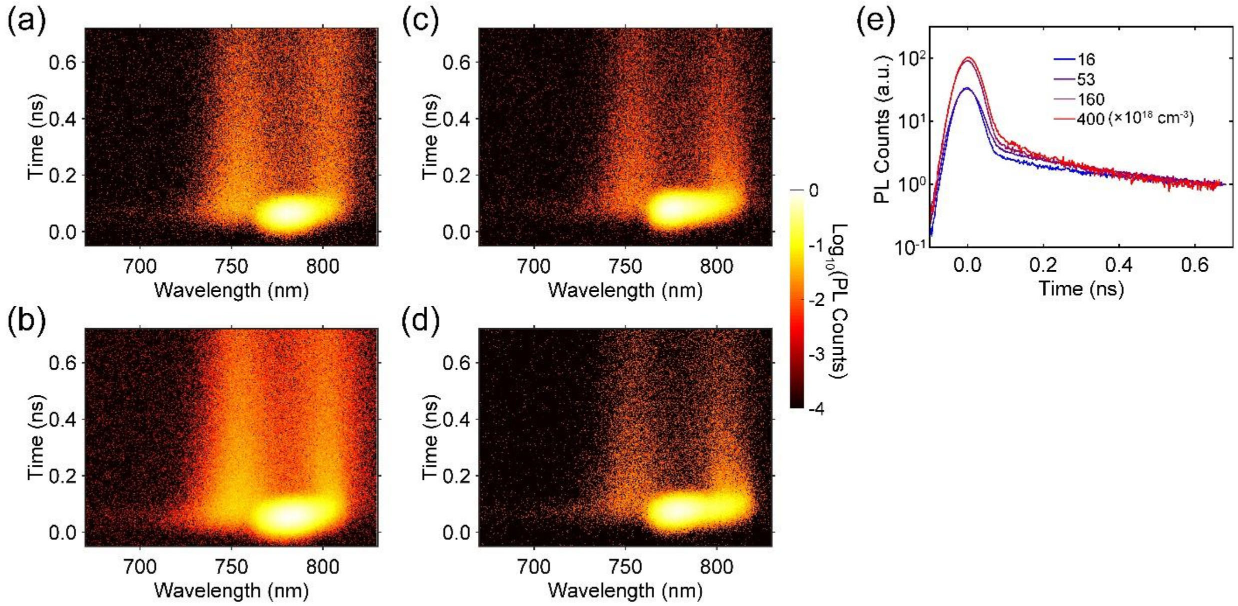

Supplementary Figure 12. (a) to (d) Time-resolved PL spectra (0~0.7 ns) for $MAPbI_3$ measured with a streak camera at 140 K under different n_0. (a) 16×10^{18} cm^{-3}; (b) 53×10^{18} cm^{-3}; (c) 160×10^{18} cm^{-3}; (d) 400×10^{18} cm^{-3}. The data shown in (a) to (d) are plotted under $\log_{10}$ scale with the corresponding scalebar shown on the right. (e) Dynamics of the PL (0~0.7 ns) under different n_0, integrated over the entire spectral window as shown in (a) to (d).

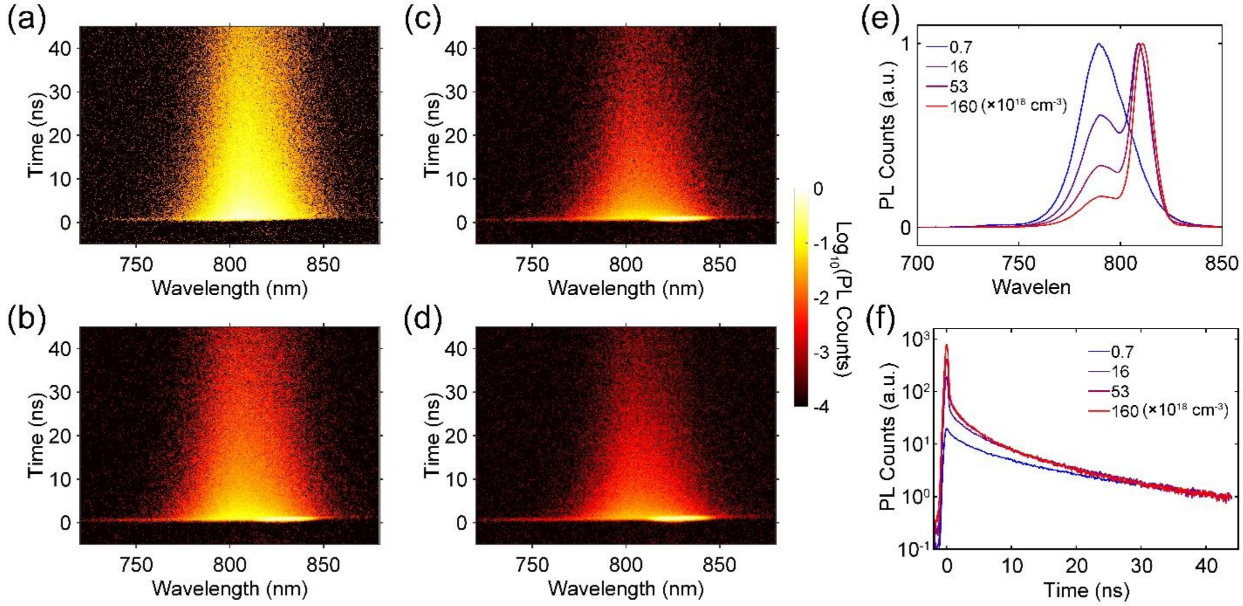

Supplementary Figure 13. (a) to (d) Time-resolved PL spectra (0~50 ns) for $MAPbI_3$ measured with a streak camera at 150 K under different n_0. (a) 0.7×10^{18} cm^{-3}; (b) 16×10^{18} cm^{-3}; (c) 53×10^{18} cm^{-3}; (d) 160×10^{18} cm^{-3}. The data shown in (a) to (d) are plotted under $\log_{10}$ scale with the corresponding scalebar shown on the right. (e) Time-integrated PL spectra measured with a CCD camera under different n_0. (f) Dynamics of the PL (0~50 ns) under different n_0, integrated over the entire spectral window as shown in (a) to (d).

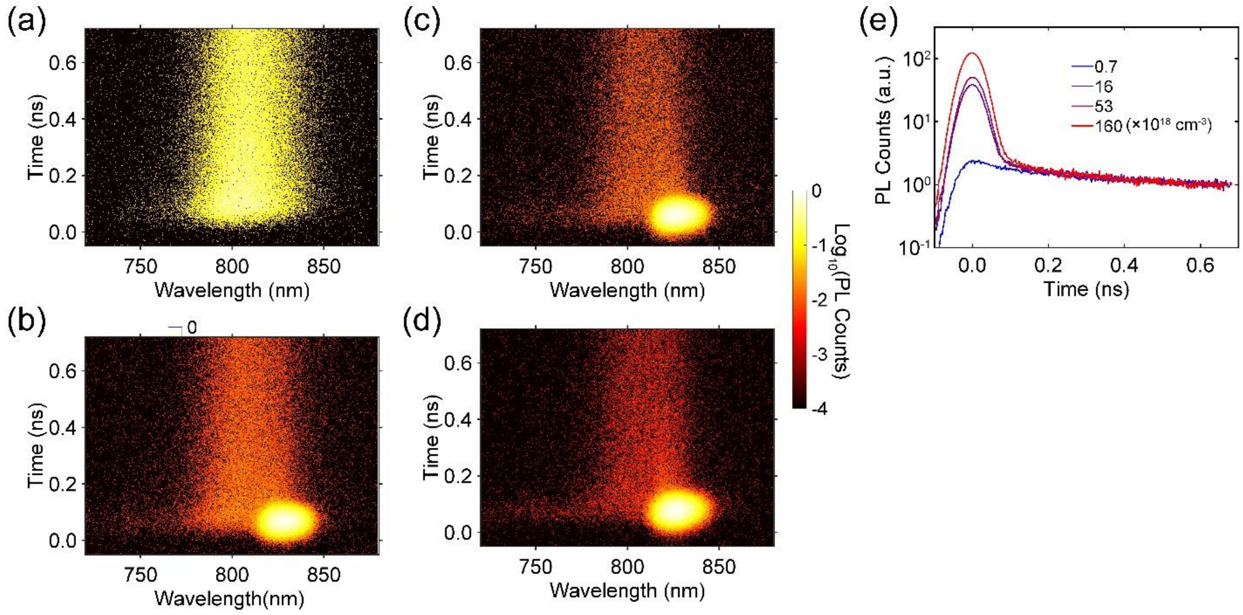

Supplementary Figure 14. (a) to (d) Time-resolved PL spectra (0~0.7 ns) for $MAPbI_3$ measured with a streak camera at 150 K under different n_0. (a) 0.7×10^{18} cm^{-3}; (b) 16×10^{18} cm^{-3}; (c) 53×10^{18} cm^{-3}; (d) 160×10^{18} cm^{-3}. The data shown in (a) to (d) are plotted under $\log_{10}$ scale with the corresponding scalebar shown on the right. (e) Dynamics of the PL (0~0.7 ns) under different n_0, integrated over the entire spectral window as shown in (a) to (d).

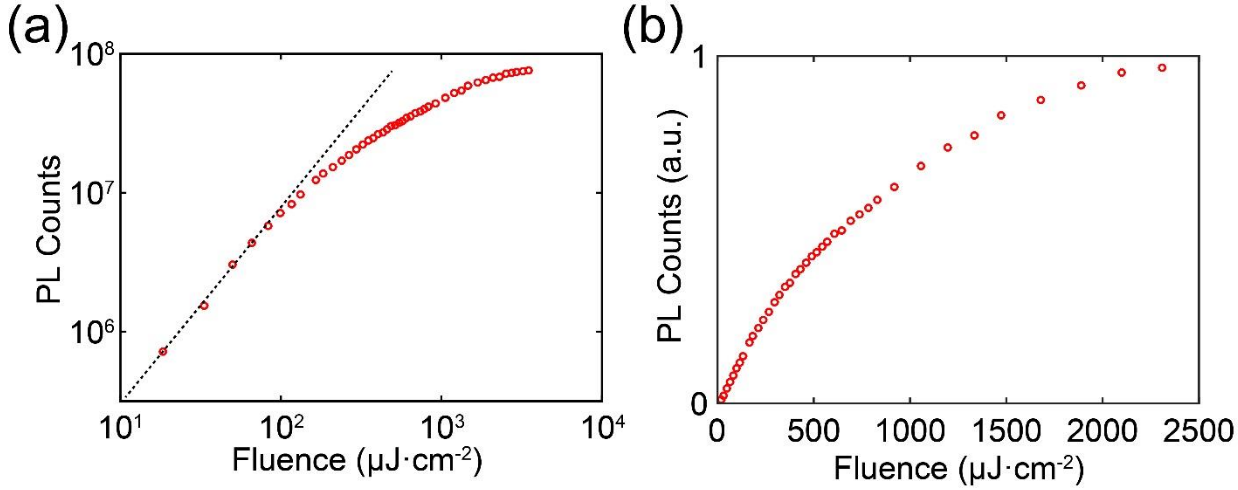

Supplementary Figure 15. Time-integrated total PL counts (measured with a CCD camera) as a function of fluence plotted in log-log scale in (a), and in linear scale in (b).

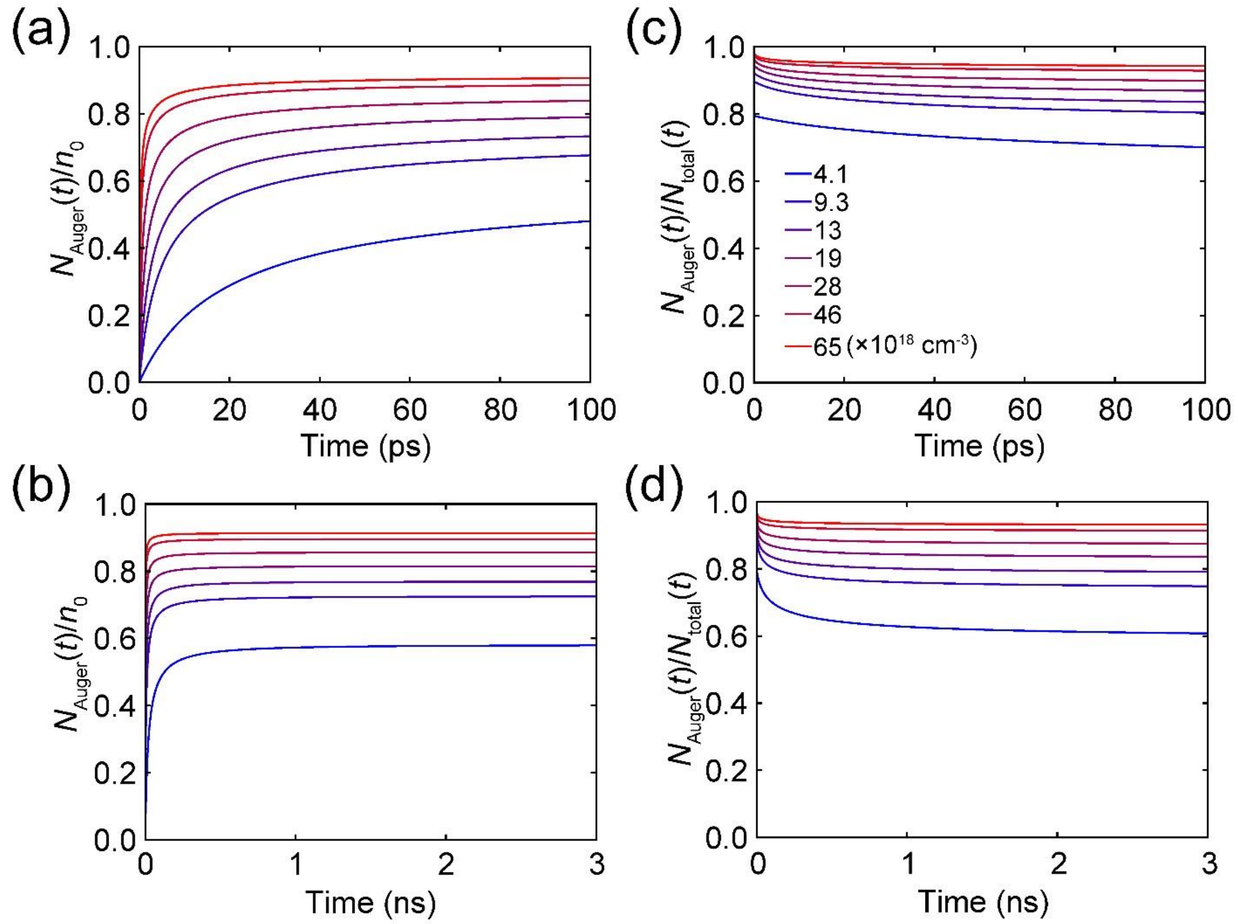

Supplementary Figure 16. Calculated carrier recombination *via* Auger process at 80 K. $N_{Auger}(t)/n_0$ plotted for 0-100 ps in (a), and 0-3 ns in (b). $N_{Auger}(t)/N_{total}(t)$ plotted for 0-100 ps in (c), and for 0-3 ns in (d). $N_{Auger}(t)$ denotes the density of carrier that have recombined *via* Auger process at time t. $N_{total}(t)$ denotes the total density of carrier that have recombined at time t, including Auger, radiative, and trap-assisted processes. The values of n_0 is shown in (c) and applies to all panels.

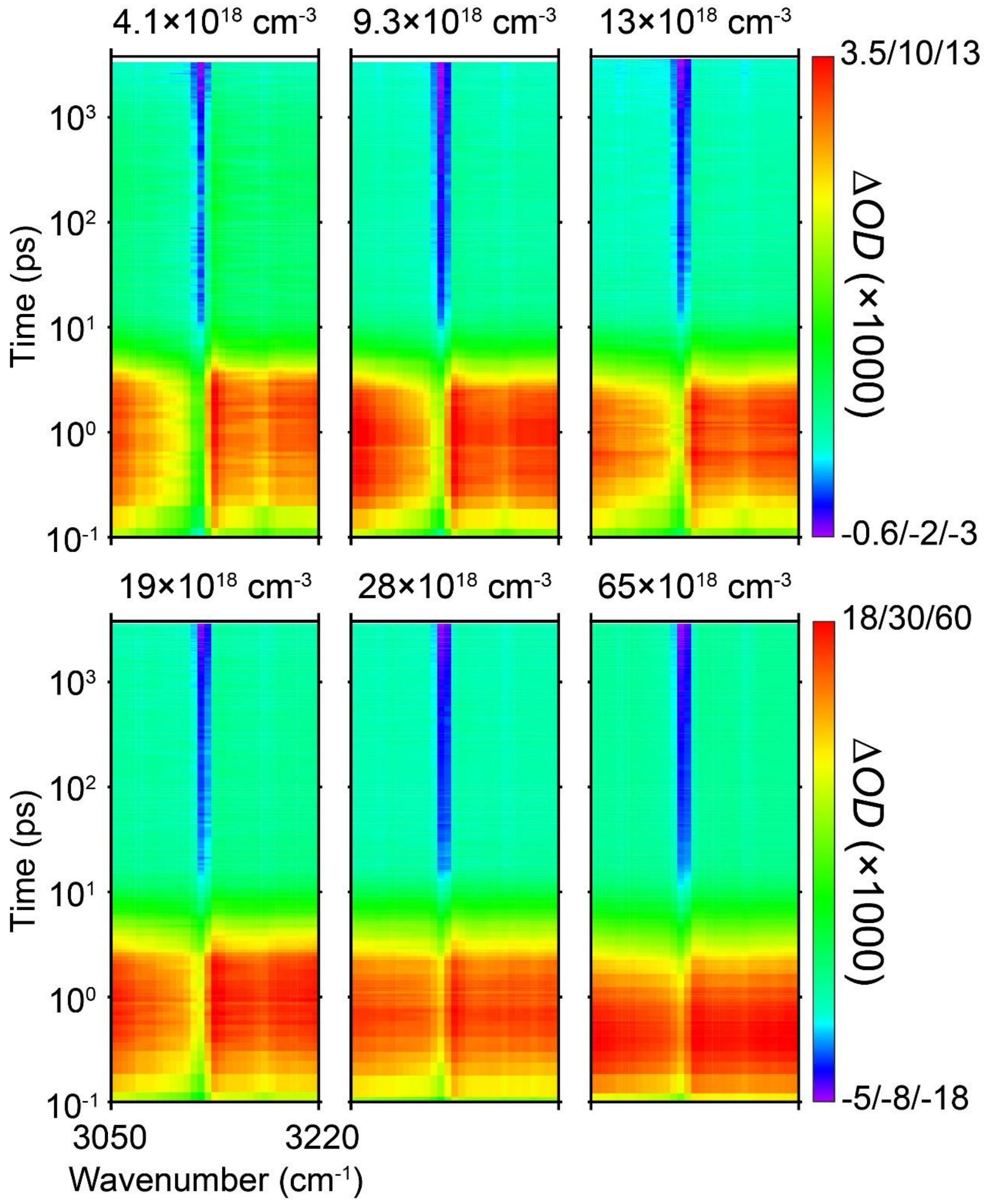

Supplementary Figure 17. Full Δ*OD* transient spectral maps of $MAPbI_3$ measured at 80 K using 500 nm pump under various n_0. The corresponding n_0 is shown on top of each spectral map.

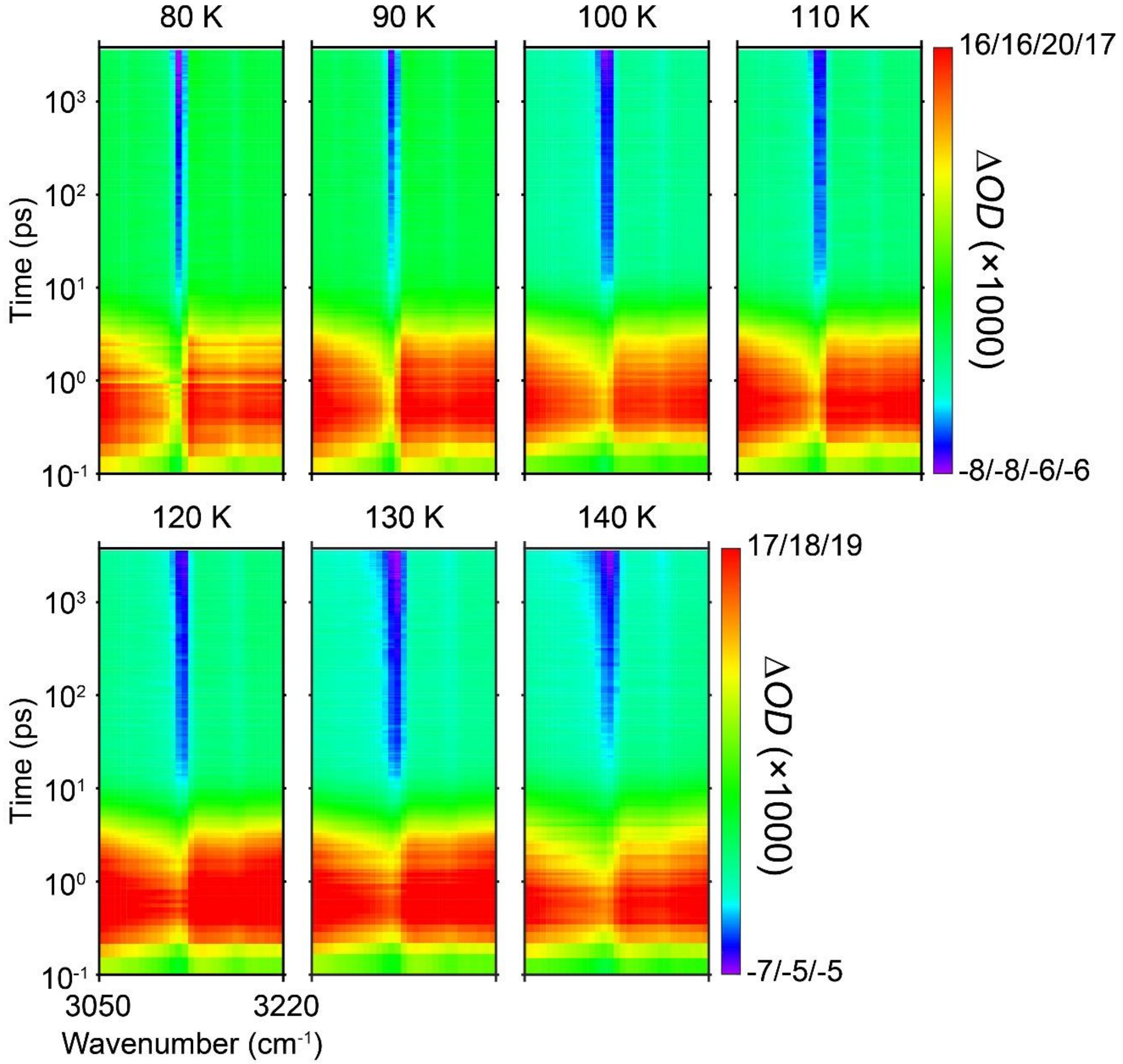

Supplementary Figure 18. Full Δ*OD* transient spectral maps of $MAPbI_3$ measured by 500 nm pump under different temperatures with n_0=35×10^{18} cm^{-3}.

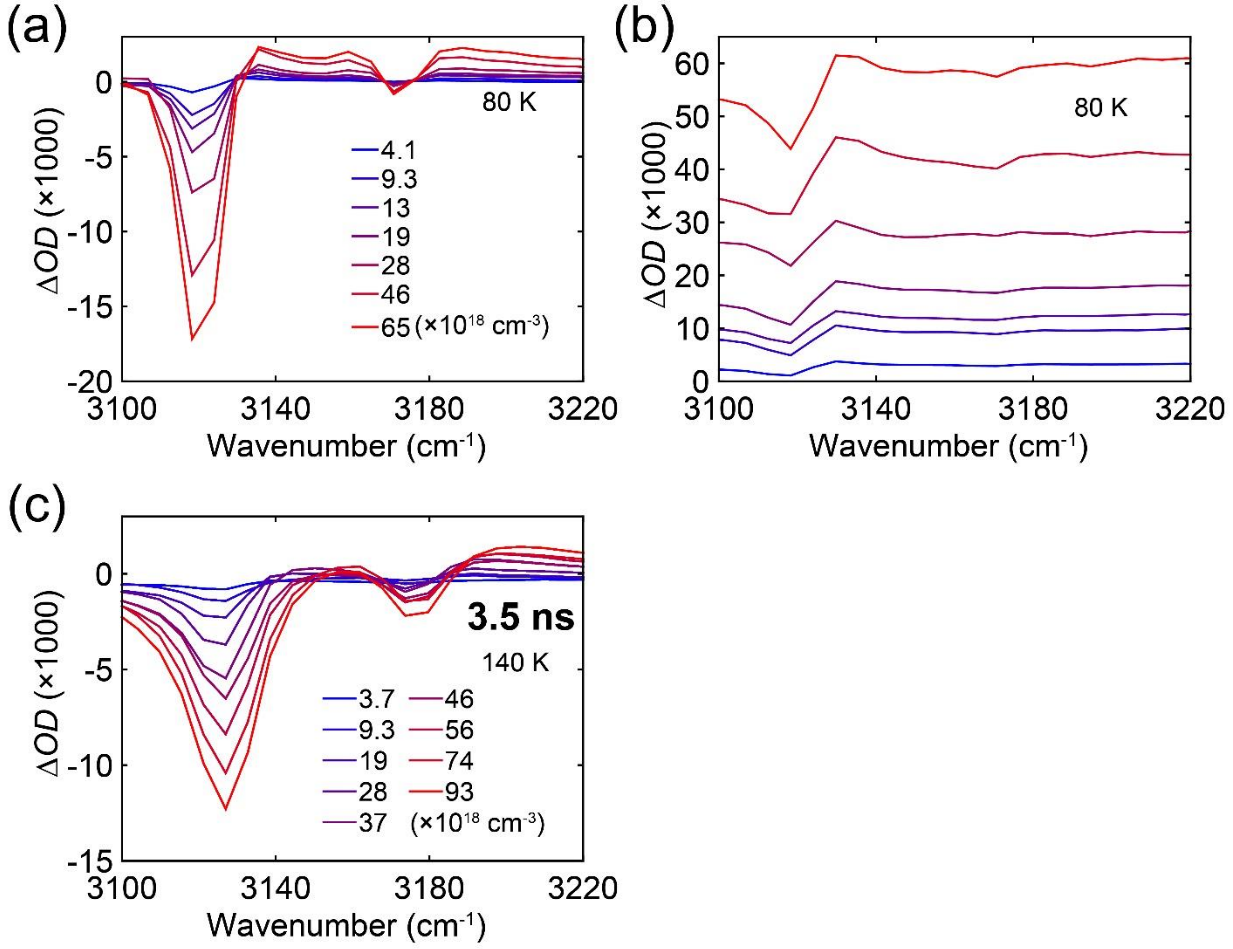

Supplementary Figure 19. Spectra of the bleaching component in (a) and PIA component in (b) for $MAPbI_3$ measured at 80 K using 500-nm pump under various n_0. The corresponding kinetics are shown in Fig. 2d and 2e, respectively, in the main text. (c) Transient spectra at delay time of 3.5 ns measured at 140 K under various n_0.

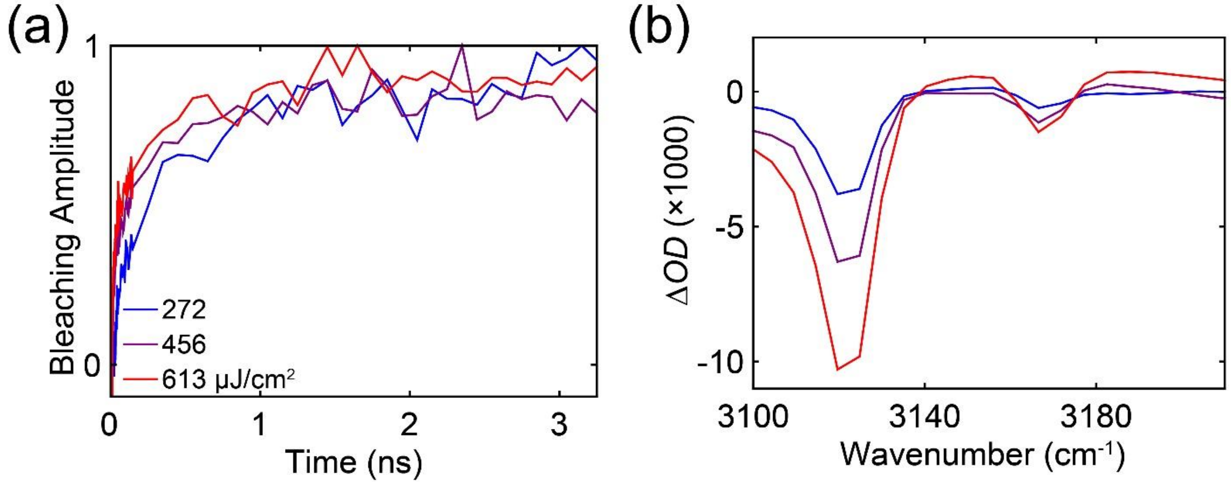

Supplementary Figure 20. (a) Kinetics and (b) Spectra of the bleaching component measured on a $MAPbI_3$ film fabricated using a different method. For the fabrication of this $MAPbI_3$ film, methylammonium iodide and PbI_2 (Sigma-Aldrich) were used as received. Film was prepared in a nitrogen-filled glovebox (H_2O and O_2 less than 1ppm) using antisolvent method as described elsewhere[3]. A 1:1 molar ratio of MAI and PbI_2 was dissolved in anhydrous N,N-dimethylformamide at 1 M concentration. The perovskite solution was then spin-coated onto the glass substrates at 3000 r.p.m. for 30 s. During spin coating, at around 6-7 s, an excess of anhydrous chlorobenzene was rapidly dropped onto the spinning film. After spin-coating, films were annealed at 100 °C for 15 min.

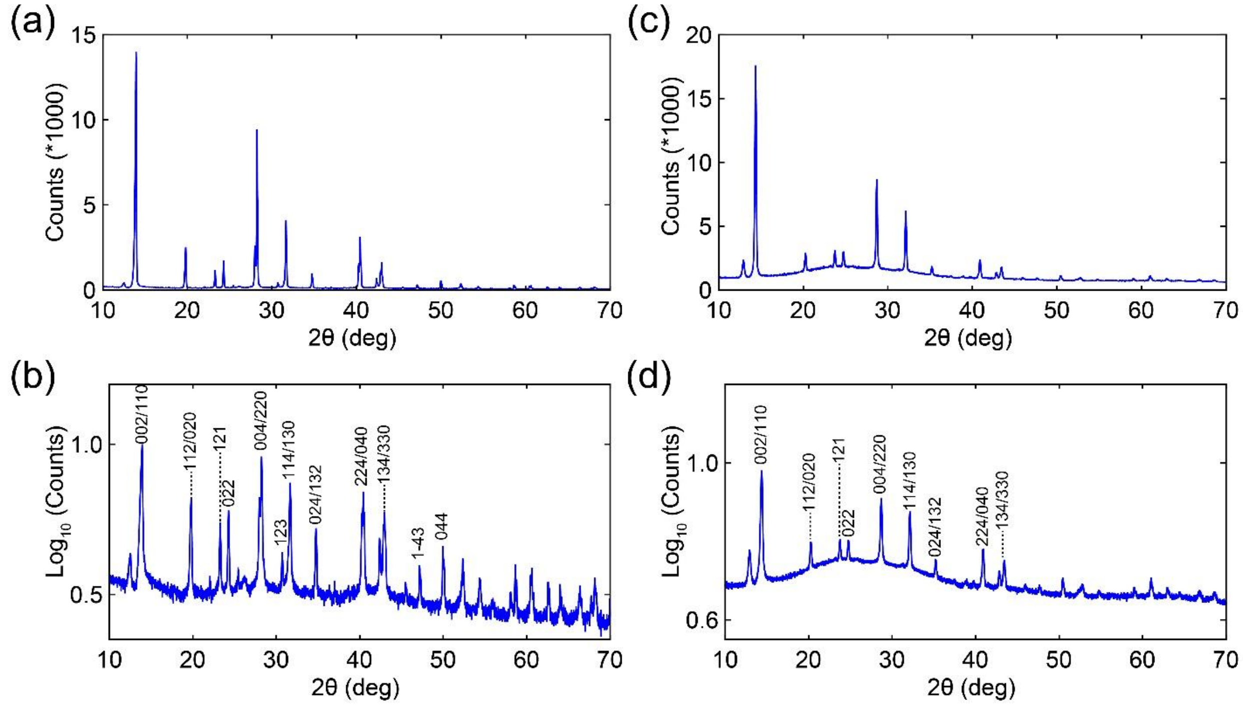

Supplementary Figure 21. X-ray diffraction pattern of the main $MAPbI_3$ sample on CaF_2 in linear scale in (a), and in $\log_{10}$ scale in (b). X-ray diffraction pattern of the additional $MAPbI_3$ sample (TA results shown in Supplementary Fig. 20) on glass in linear scale in (c), and in $\log_{10}$ scale in (d). The background signals in (c) and (d) are due to the glass substrate.

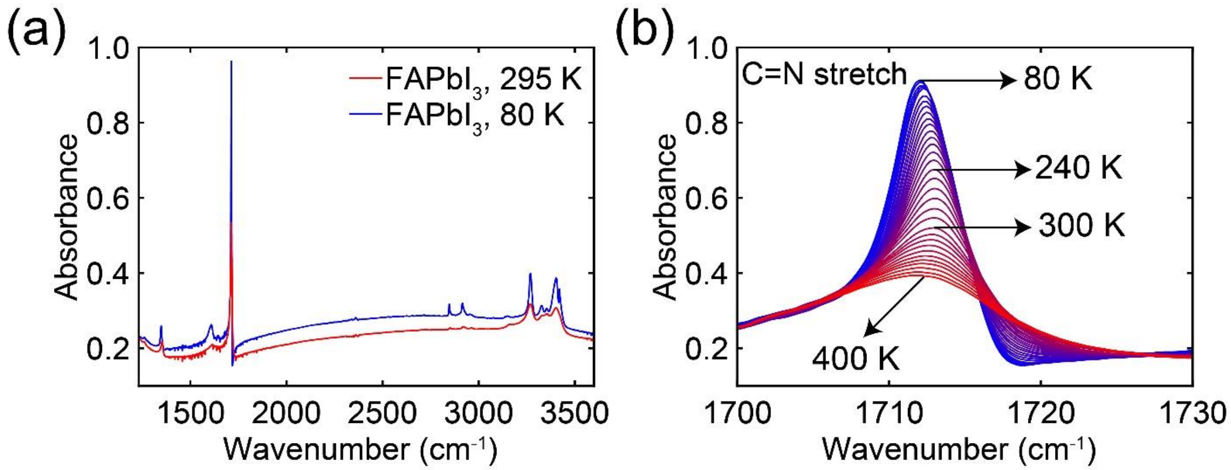

Supplementary Figure 22. Infrared absorbance spectrum of $FAPbI_3$. (a) Spectra taken at 295 K and 80 K. (b) Spectra from 1700 to 1730 cm^{-1} taken at 80 K (blue) to 400 K (red) in increment of 10 K.

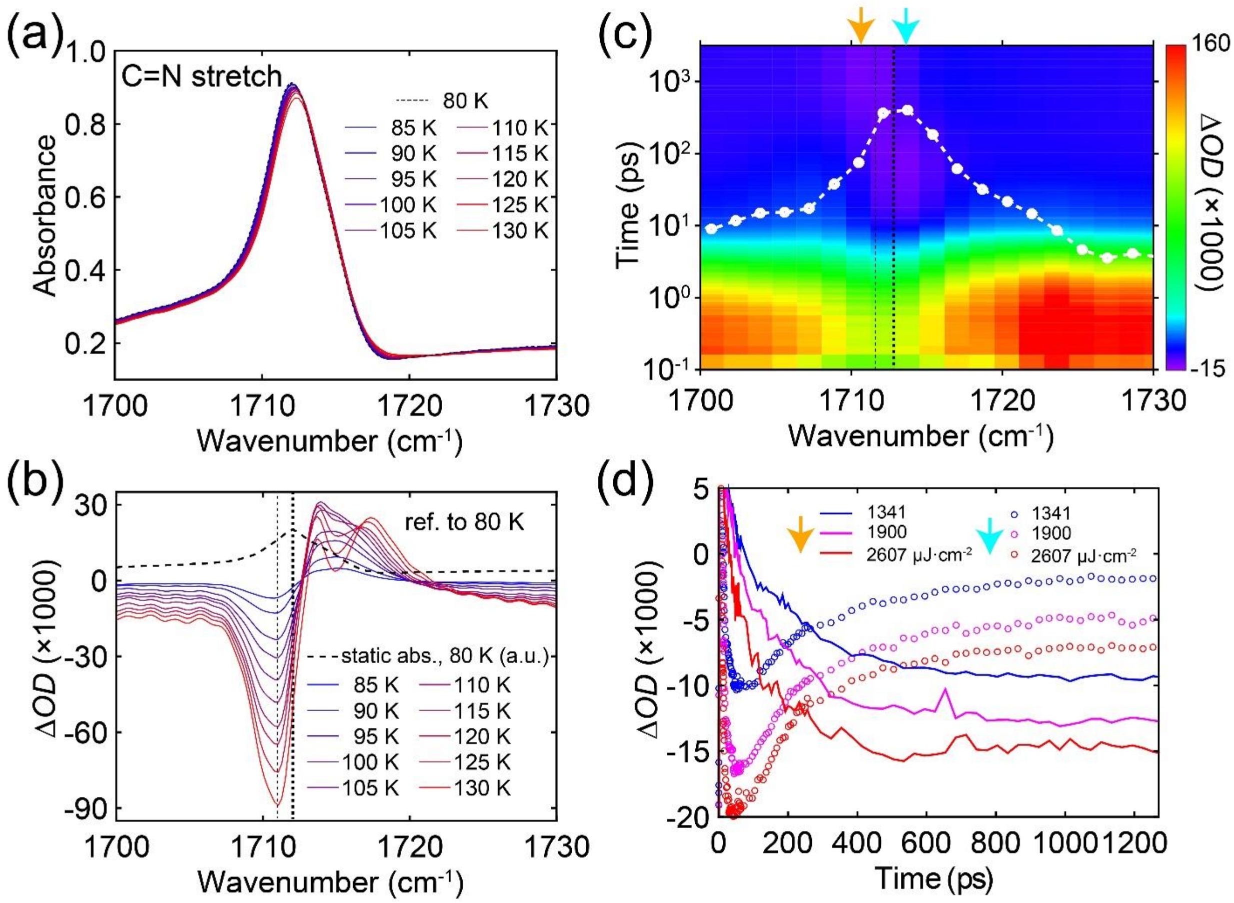

Supplementary Figure 23. Static and transient absorption characterization of the C=N stretching mode of $FAPbI_3$. (a) Static temperature dependent absorbance and (b) Differential absorption spectra (referenced to 80 K) of the C=N stretching mode. (c) Transient spectral map around the C=N stretching mode taken at 80 K using 500 nm pump excitation (1341 μJ·cm^{-2} fluence). White-dashed line indicates static absorbance of the probe measured using the array detector (circles indicate pixels of detector). Thick and thin dashed lines highlight the center wavelengths of the early- and late-time bleach dips. (d) Kinetic traces plotted for wavelengths marked in (c) by orange and cyan arrows under various fluences.

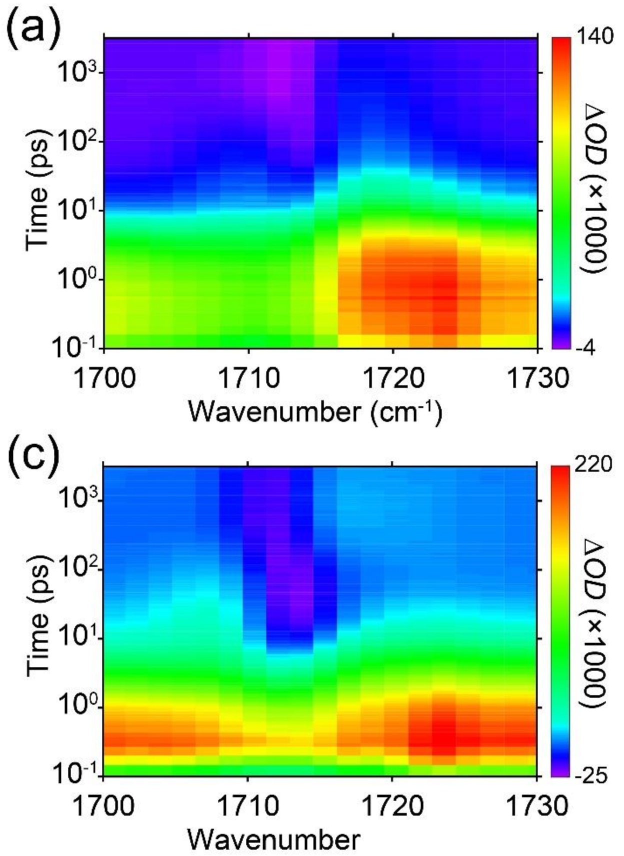

Supplementary Figure 24. Full Δ*OD* transient spectral maps measured on $FAPbI_3$ at 80 K using 500 nm pump under fluences of 710, 1900 and 2607 μJ·cm^{-2} shown in (a), (b) and (c).

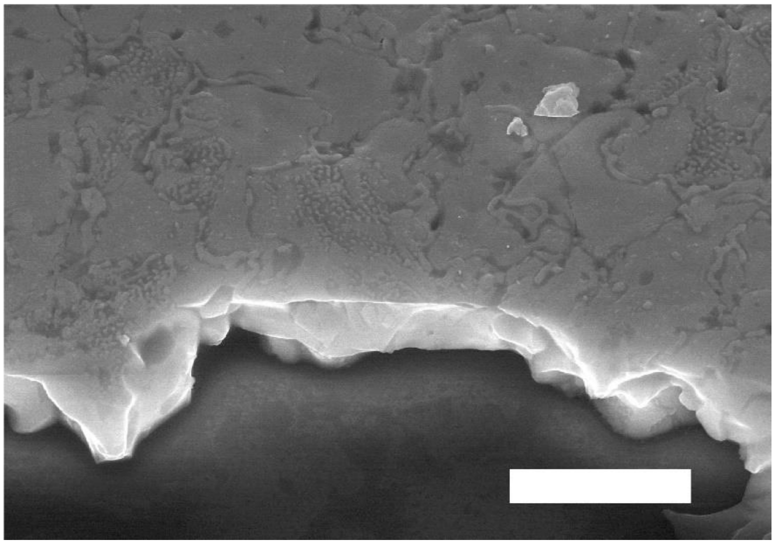

Supplementary Figure 25. SEM image of the $MAPbI_3$ thin film. The scalebar is 5 µm.

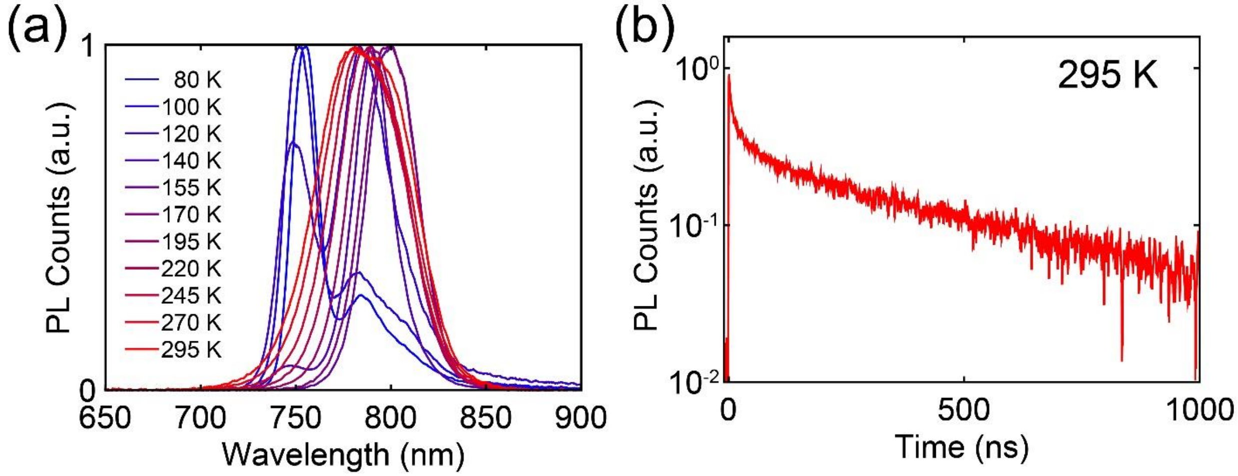

Supplementary Figure 26. (a) Time-integrated PL spectra of the $MAPbI_3$ film from 295 K to 80 K. (b) Time-resolved photoluminescence trace (at the PL peak wavelength) of the $MAPbI_3$ film at 295 K. Time-integrated data in (a) was acquired by exciting the sample at 400 nm with the frequency-doubled titanium:sapphire amplifier output, with fluence corresponding to a carrier density less than 1×10^{18} cm^{-3}. Time-resolved data in (b) was obtained using time-correlated single-photon-counting electronics with the sample excited at 405 nm (fluence was ~ 40 nJ/cm^2) by a 35-ps pulse-width laser diode.

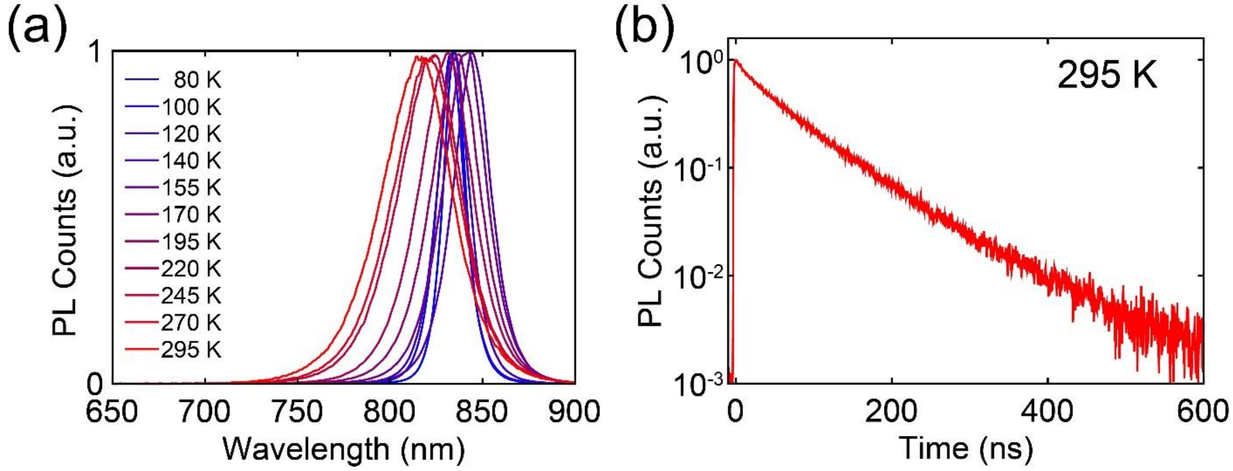

Supplementary Figure 27. (a) Time-integrated photoluminescence spectra of the $FAPbI_3$ film from 295 K to 80 K. (b) Time-resolved photoluminescence decay (at the PL peak wavelength) of the $FAPbI_3$ film at 295 K. Time-integrated data in (a) was acquired by exciting the sample at 400 nm with the frequency-doubled titanium:sapphire amplifier output, with fluence corresponding to a carrier density less than 1×10^{18} cm^{-3}. Time-resolved data in (b) was obtained using time-correlated single-photon-counting electronics with the sample excited at 405 nm (fluence was ~ 40 nJ/cm^2) by a 35-ps pulse-width laser diode.

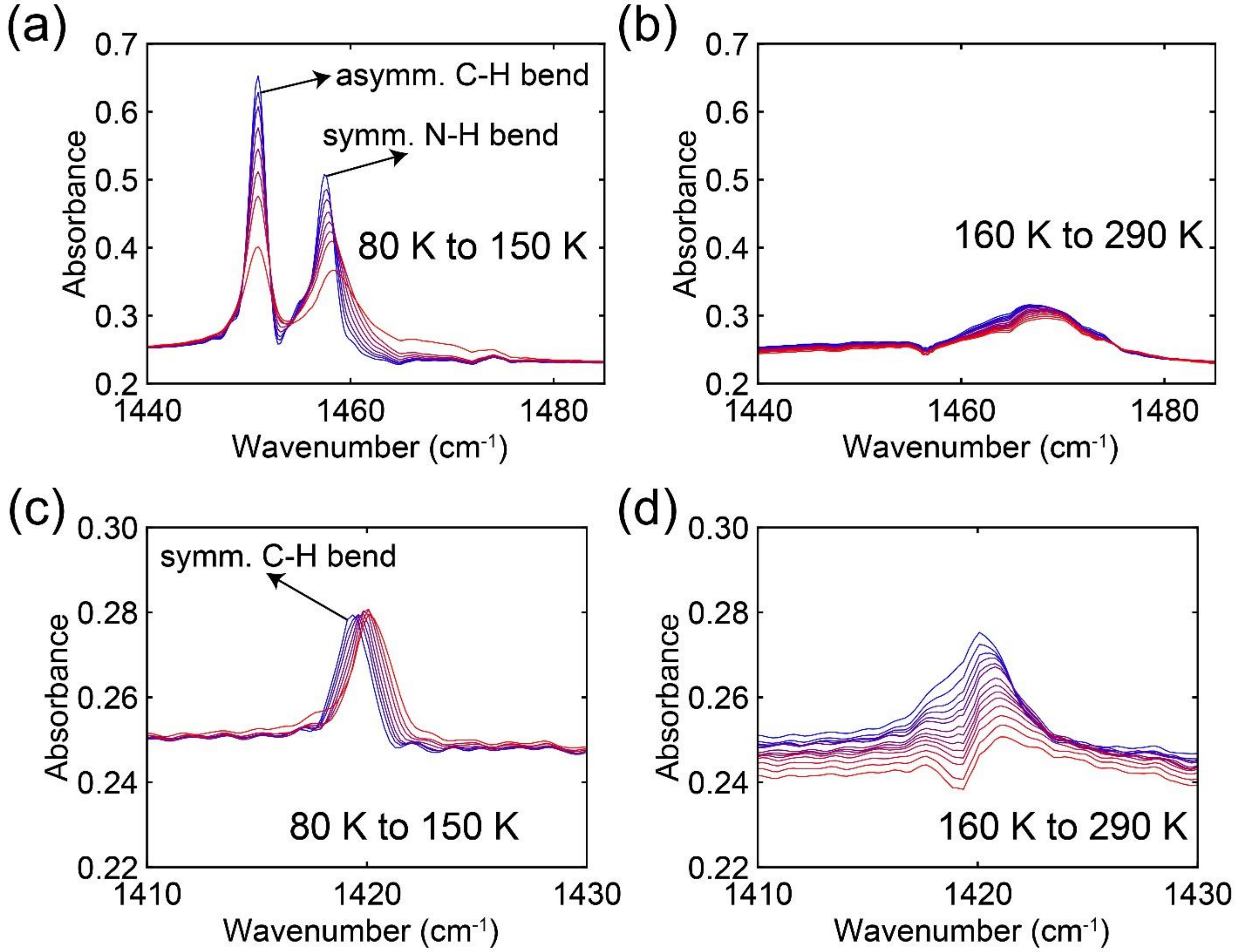

Supplementary Figure 28. Absorbance of the asymmetric C-H and symmetric N-H bending modes of $MAPbI_3$ from 80 K to 150 K in (a), and from 160 K to 290 K in (b), both in increments of 10 K. Absorbance of the symmetric C-H bending mode of $MAPbI_3$ from 80 K to 150 K in (c), and from 160 K to 290 K in (d), both in increments of 10 K. Assignment of the peaks are according to reference[4].

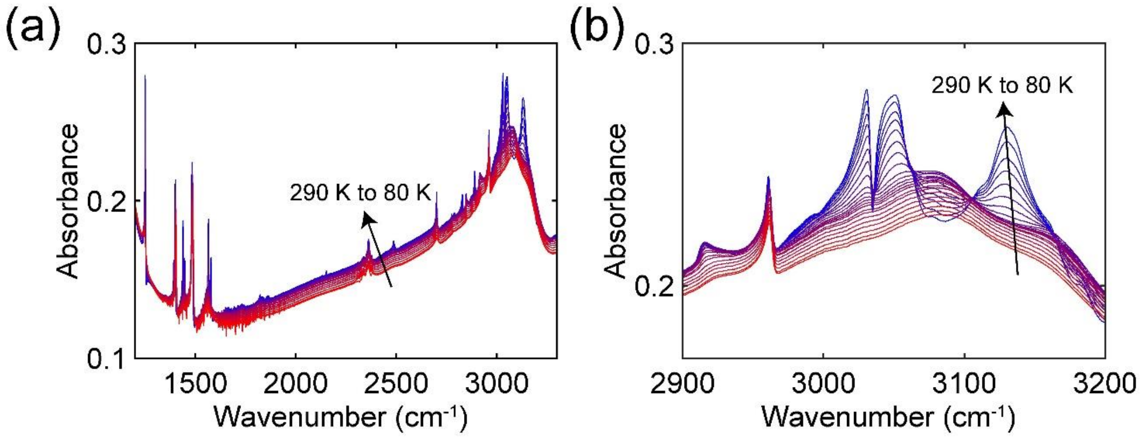

Supplementary Figure 29. Infrared absorbance of an MAI film from 290 K (red) to 80 K (blue) in decrements of 10 K. (a) From 1200 to 3300 cm^{-1}. (b) From 2900 to 3200 cm^{-1}. The N-H stretching modes of the MAI film in the 3000 to 3200 cm^{-1} range exhibit strong temperature dependence, especially at low temperature range (<160 K). Different from $MAPbI_3$ which has a tetragonal-to-orthorhombic phase transition at 162 K, the variation of intensity is continuous upon cooling for the MAI film.

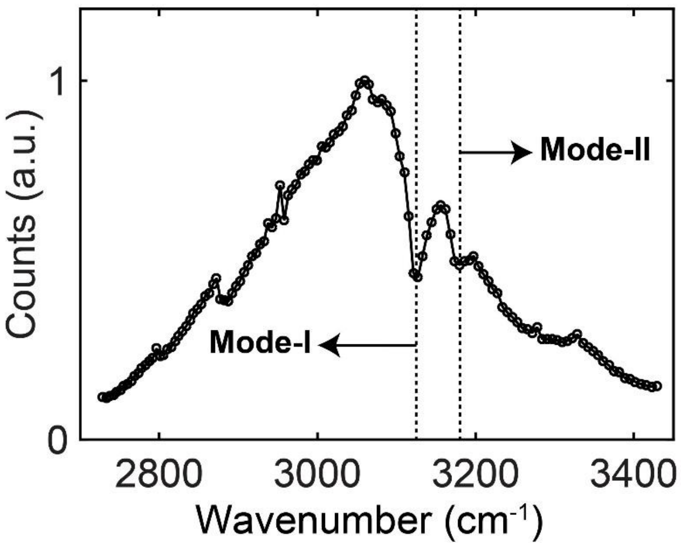

Supplementary Figure 30. Typical spectrum of the mid-infrared probe captured by the array detector (with the $MAPbI_3$ thin film in the beam).

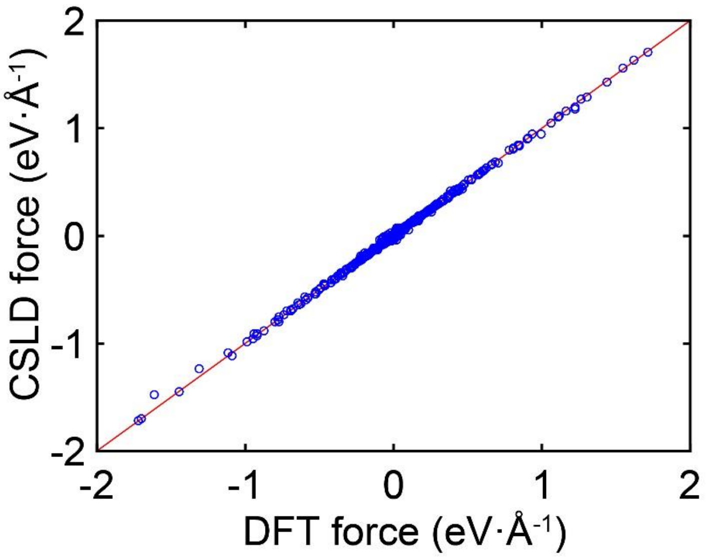

Supplementary Figure 31. Comparison between forces predicted by compressive sensing lattice dynamics (CSLD) and those directly computed from DFT. The red line (x=y) indicates where the results are identical.

Supplementary Note 1. Fitting of the film thickness

As shown in Supplementary Fig. 1a, the thickness of the $MAPbI_3$ film used in this work gives rise to several Fabry-Perot-type transmission dips in the near- to mid-infrared range. Matching the calculated transmission dips (using transfer-matrix method) with the measured ones yields an estimated film thickness of 680 nm. As the spectral locations of the dips are determined only by Re(ε) but not Im(ε), we modeled $MAPbI_3$ as a dielectric material with Re(ε)=4.85 and Im(ε)=0 (references 1 and 2).

Supplementary Note 2. Calculation of the carrier density

The initial carrier density (n_0) after pump excitation was estimated as $n_0 = \frac{F(1-R)(1-1/e)}{\hbar\omega \cdot l}$, where l is the penetration depth of the pump (taken to be $1/\alpha$), F is the pump fluence (measured outside the cryostat), $\hbar\omega$ is the photon energy (2.48 eV) of the pump, and e is the natural logarithm constant. The absorption coefficient α at 500 nm is 1.11×10^5 cm^{-1} (reference[2]). The total reflection from the cryostat window and from the sample, R, is ~20%. Supplementary Fig. 4 shows the dependence of n_0 on the pump fluence.

Supplementary Note 3. Derivative-like features in the photoinduced absorption spectra

We used two Lorentz oscillators to model the dielectric constants of $MAPbI_3$ around the N-H-stretching modes (mode-I and mode-II) at 80 K. The relative permittivity was written as $\varepsilon(\omega) = \varepsilon_\infty + \frac{{\omega_{01}}^2}{{\omega_1}^2-\omega^2-i\gamma_1\omega} + \frac{{\omega_{02}}^2}{{\omega_2}^2-\omega^2-i\gamma_2\omega}$, where ε_∞ was taken as 4.85. The film transmission, reflection and absorption were calculated using a transfer-matrix code. Best match (shown in Supplementary Fig. 6a) between measured and calculated absorbance spectra was obtained with $\omega_{01}=2.9\times10^{13}$ rad·s^{-1}, $\omega_1=5.986\times10^{14}$ rad·s^{-1}, $\gamma_1=3.2\times10^{12}$ rad·s^{-1}, $\omega_{02}=5.0\times10^{13}$ rad·s^{-1}, $\omega_2=5.888\times10^{14}$ rad·s^{-1}, $\gamma_2=3\times10^{12}$ rad·s^{-1}. The resulting imaginary and real components of relative permittivity, denoted as Re(ε) and Im(ε), are plotted in Supplementary Figs. 6b and 6c, respectively.

The measured broadband photoinduced absorption (PIA) at 0.1 ps delay time is shown in Supplementary Fig. 6d. Note that a derivative-like feature appears in the PIA spectrum, with a dip and peak on the red and blue side of the late-time bleaching dips; the latter spectrally match the static absorption peaks and indicate lattice heating (see Fig. 2b). As we find that the

amplitude of PIA increases at lower wavenumbers, to qualitatively capture such behavior we introduced an asymmetric, positive ΔIm(ε), which has a larger amplitude at lower wavenumber (Supplementary Fig. 7a). Such change of ΔIm(ε) with no peaks in the considered spectral range cannot result in any resonant, or derivative-like spectral features in the PIA spectrum. However, the Kramers-Kronig relationship dictates that ΔIm(ε) induces a corresponding ΔRe(ε) calculated as $\Delta Re[\varepsilon(\omega)] = \frac{2}{\pi}\mathbf{P}\int_0^\infty d\omega_0 \frac{\omega_0 \Delta \mathrm{Im}[\varepsilon(\omega_0)]}{{\omega_0}^2 - \omega^2}$, where **P** denotes the principle value of the integral. The calculated ΔRe(ε) is shown in Supplementary Fig. 7a, and Re(ε) and Im(ε) before and after the changes are shown in Supplementary Fig. 7b. The calculated change of film absorbance due to the change of both Re(ε) and Im(ε) plotted in Supplementary Figs. 7c and 7d, using the transfer-matrix method, shows the derivative-like features in the PIA spectrum. Note that the Kramers-Kronig calculation presented here is only intended to qualitatively explain the origin of the derivative-like feature in the PIA component in our TA experiments. Fully quantitative modeling of the change of Re(ε) and Im(ε) requires the knowledge of the entire spectral response from zero to infinity frequency, which is beyond the scope of the work.

Supplementary Note 4. Ultrafast Auger heating

We calculated the timescales of Auger, radiative and trap-assisted recombination at 80 K using the rate equation for carrier recombination, $-\frac{dn(t)}{dt} = An(t) + Bn(t)^2 + Cn(t)^3$. The recombination rate constants, *A*, *B* and *C*, were taken from earlier work[5]. In the calculations, we used the same initial carrier density (n_0) as shown in Fig. 2d in the main text. Note that these calculated densities are upper bounds on the true densities, because (i) we cannot eliminate the possibility of saturable absorption; (ii) a significant portion of excited carriers undergo amplified spontaneous emission, as shown in the time-resolved PL spectra (Supplementary Figs. 8 to 14).

The plots of $N_{\mathrm{Auger}}(t)/n_0$ and $N_{\mathrm{Auger}}(t)/N_{\mathrm{total}}(t)$ are presented in Supplementary Fig. 16. N_{Auger} denotes the concentrations of carriers that have recombined *via* Auger process at time *t*. As shown in Supplementary Fig. 16, in the relevant range of n_0, Auger process takes place in a few tens of picoseconds, which is significantly shorter than the nanosecond timescale of the bleaching component, indicating that the slow rise of the bleaching component cannot arise from Auger heating.

Supplementary Note 5. Transient response of the C=N stretching mode of $FAPbI_3$

The static spectra (Supplementary Fig. 23a) demonstrate a strong absorption feature centered at 1712 cm^{-1} owing to the C=N stretching mode. Upon heating, the C=N stretching mode decreases in intensity, and blueshifts. Hence, the corresponding bleaching dip in the differential absorption spectra (Supplementary Fig. 23b) at a higher temperature is redder than the absorption peak at lower temperature. Resolution of such a fine spectral shift is owing to an extremely narrow FWHM of 5 cm^{-1} (as compared to mode-I in the orthorhombic phase of $MAPbI_3$ with ~17 cm^{-1} FWHM). A transient spectral map (Supplementary Fig. 23c) acquired using 500 nm excitation reveals a bleaching feature indicative of heating of the lattice. However, only at late delay times (later than hundreds of ps) is the bleach feature residing on the red side of the absorption peak, which is expected for the thermally equilibrated lattice according to the static differential spectra (Supplementary Fig. 23b). Kinetic traces at the center wavelengths of the early-time and late-time bleaching features plotted in Supplementary Fig. 23d demonstrate a few-hundred-ps timescale for the spectral redshift, which shows negligible fluence dependence. Therefore, thermal equilibration time inferred from the transient response of C=N stretching mode of $FAPbI_3$ is within the same order-of-magnitude as the thermal equilibration time in $MAPbI_3$.

Supplementary References

1. T. Glaser *et al.*, Infrared spectroscopic study of vibrational modes in methylammonium lead halide perovskites. *J. Phys. Chem. Lett.* **6**, 2913-2918 (2015).
2. A. M. A. Leguy *et al.*, Experimental and theoretical optical properties of methylammonium lead halide perovskites. *Nanoscale* **8**, 6317-6327 (2016).
3. M. Xiao *et al.*, A fast deposition-crystallization procedure for highly efficient lead iodide perovskite thin-film solar cells. *Angew. Chem.* **126**, 10056-10061 (2014).
4. M. A. Pérez-Osorio *et al.*, Vibrational properties of the organic–inorganic halide perovskite $CH_3NH_3PbI_3$ from theory and experiment: factor group analysis, first-principles calculations, and low-temperature infrared spectra. *J. Phys. Chem. C* **119**, 25703-25718 (2015).
5. R. L. Milot, G. E. Eperon, H. J. Snaith, M. B. Johnston, L. M. Herz, Temperature-dependent charge-carrier dynamics in $CH_3NH_3PbI_3$ perovskite thin films. *Adv. Funct. Mater.* **25**, 6218-6227 (2015).
